# Supplementary material for: Deep (phospho)proteomics profiling of pre- treatment needle biopsies identifies signatures of treatment resistance in HER2+ breast cancer
Source: Cell Rep Med. 2023 Oct 3;4(10):101203. doi: 10.1016/j.xcrm.2023.101203 (PMC10591042; doi:10.1016/j.xcrm.2023.101203)
Supplement: Document S1. Table S1 and Figures S1–S6 [file mmc1.pdf]

Cell Reports Medicine, Volume *4*

Supplemental information

Deep (phospho)proteomics profiling of pre-treatment needle biopsies identifies signatures of treatment resistance in $HER2^+$ breast cancer

Donna O. Debets, Kelly E. Stecker, Anastasia Piskopou, Marte C. Liefaard, Jelle Wesseling, Gabe S. Sonke, Esther H. Lips, and Maarten Altelaar

Supplementary Table 1. Patient information. Related to STAR Methods.

Patient	*Histology*	*Tumour grade*	*ER (%)*	*PR (%)*	*HER2 score*	*Treatment outcome*	*Tumour (%)*	*TMT pool_label*
1	ID	2	50	0	3+	pCR	80	Pool1_126
2	ID	2	80	80	3+	pCR	80	Pool1_127N
3	ID	3	0	0	3+	pCR	60	Pool1_127C
4	ID	2	80	5	3+	pCR	70	Pool1_128N
5	ID	2	25	0	3+	pCR	70	Pool1_128C
6	ID	3	0	0	3+	pCR	70	Pool1_129N
7	ID	3	100	1	2+	No pCR	80	Pool1_129C
8	ID	2	70	70	3+	pCR	60	Pool1_130N
9	ID	3	60	70	3+	pCR	80	Pool1_130C
10	ID	3	100	5	3+	No pCR	80	Pool1_131N
11	ID	2	100	70	3+	pCR	80	Pool2_126
12	ID	3	20	0	3+	pCR	80	Pool2_127N
13	IL	3	100	40	3+	npCR	80	Pool2_127C
14	ID	2	40	0	3+	pCR	60	Pool2_128N
15	ID	2	0	0	3+	pCR	70	Pool2_128C
16	ID	2	0	0	3+	pCR	60	Pool2_129N
17	ID	2	100	0	3+	npCR	70	Pool2_129C
18	ID	3	60	5	2+	npCR	60	Pool2_130N
19	ID	2	100	100	3+	pCR	80	Pool2_130C
20	IL	2	100	0	2+	No pCR	80	Pool2_131N
21	ID	2	100	0	3+	pCR	80	Pool3_126
22	ID	3	100	0	2+	pCR	80	Pool3_127N
23	ID	2	100	1	2+	pCR	70	Pool3_127C
24	ID	3	80	70	3+	npCR	70	Pool3_128N
25	ID	2	0	0	3+	pCR	60	Pool3_128C
26	ID	3	0	0	3+	pCR	60	Pool3_129N
27	IL	3	90	0	3+	pCR	80	Pool3_129C
28	ID	3	90	100	3+	npCR	70	Pool3_130N
29	ID	3	100	60	2+	No pCR	80	Pool3_130C
30	ID	2	90	100	3+	pCR	90	Pool4_126
31	ID	3	90	40	3+	pCR	60	Pool4_127C
32	ID	3	10	0	2+	pCR	80	Pool4_128N
33	ID	2	100	100	3+	pCR	70	Pool4_128C
34	MA	2	100	30	3+	pCR	80	Pool4_129N
35	ID	2	0	0	3+	pCR	60	Pool4_129C
36	ID	3	0	0	3+	npCR	60	Pool4_130N
37	ID	3	100	20	3+	No pCR	80	Pool5_126
38	ID	2	50	100	3+	No pCR	70	Pool5_127N
39	ID	3	100	5	3+	No pCR	80	Pool5_127C
40	ID	2	100	90	3+	npCR	60	Pool5_128N
41	ID	3	100	90	3+	No pCR	70	Pool5_128C
42	MA	2/3	90	20	3+	No pCR	60	Pool5_129N
43	ID	3	100	0	3+	No pCR	80	Pool5_129C
44	ID	2	100	40	3+	No pCR	60	Pool5_130N
45	ID	3	100	1	3+	npCR	60	Pool5_131N

ID: invasive ductal
IL: invasive lobular
MA: Mucinous adenocarcinoma

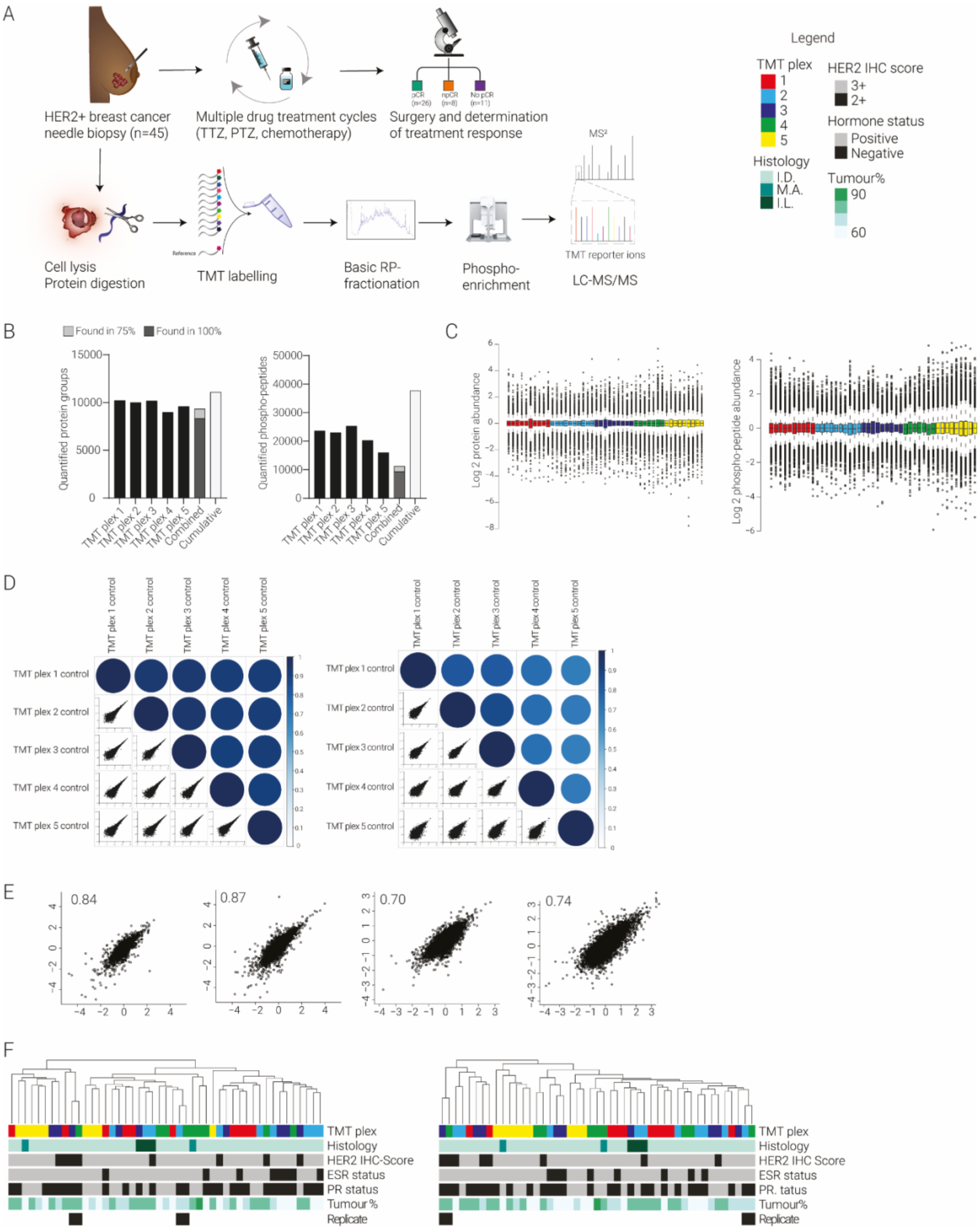

Supplementary Figure 1. Experimental workflow and data quality. Related to STAR Methods. A) 45 HER2+ breast cancer biopsies were collected by needle biopsy procedure. Afterwards, patients received a combination of targeted therapy (Trastuzumab and Pertuzumab) and chemotherapy, followed by surgery. Treatment response was determined based on the absence or presence of remaining tumour cells in the breast and lymph node. Patients were

classified as pCR (pathological complete response), npCR (near pathological complete response) or No pCR (no pathological complete response). The treatment-naive biopsies were used for the (phospho)proteomics analysis. Tumour cells were lysed, proteins extracted and digested. The derived peptides were labelled by Tandem Mass Tag (TMT). Peptides were fractionated by basic reversed-phase chromatography and phosphorylated peptides were enriched prior to LC-MS/MS analysis. B) Number of protein groups (left) and phopsho-peptides (right) quantified. C) Boxplot of median normalised samples of the protein data (left) and phospho-data (right). D) Correlation plot of the inter-plex TMT reference sample of the Log 2 protein abundance data (left) and Log 2 phosphopeptide abundance data (right). E) Correlation of replicate patient samples (Pearson correlation) of protein (left two plots) and phospho-data (right two plots). Scales represent Log2 abundances normalised to the pooled reference channel. F) Unsupervised clustering of all proteins (left) or all phosphopeptides (right) showed grouping of replicate samples. Grouping was not driven by tumour% or TMT-plex.

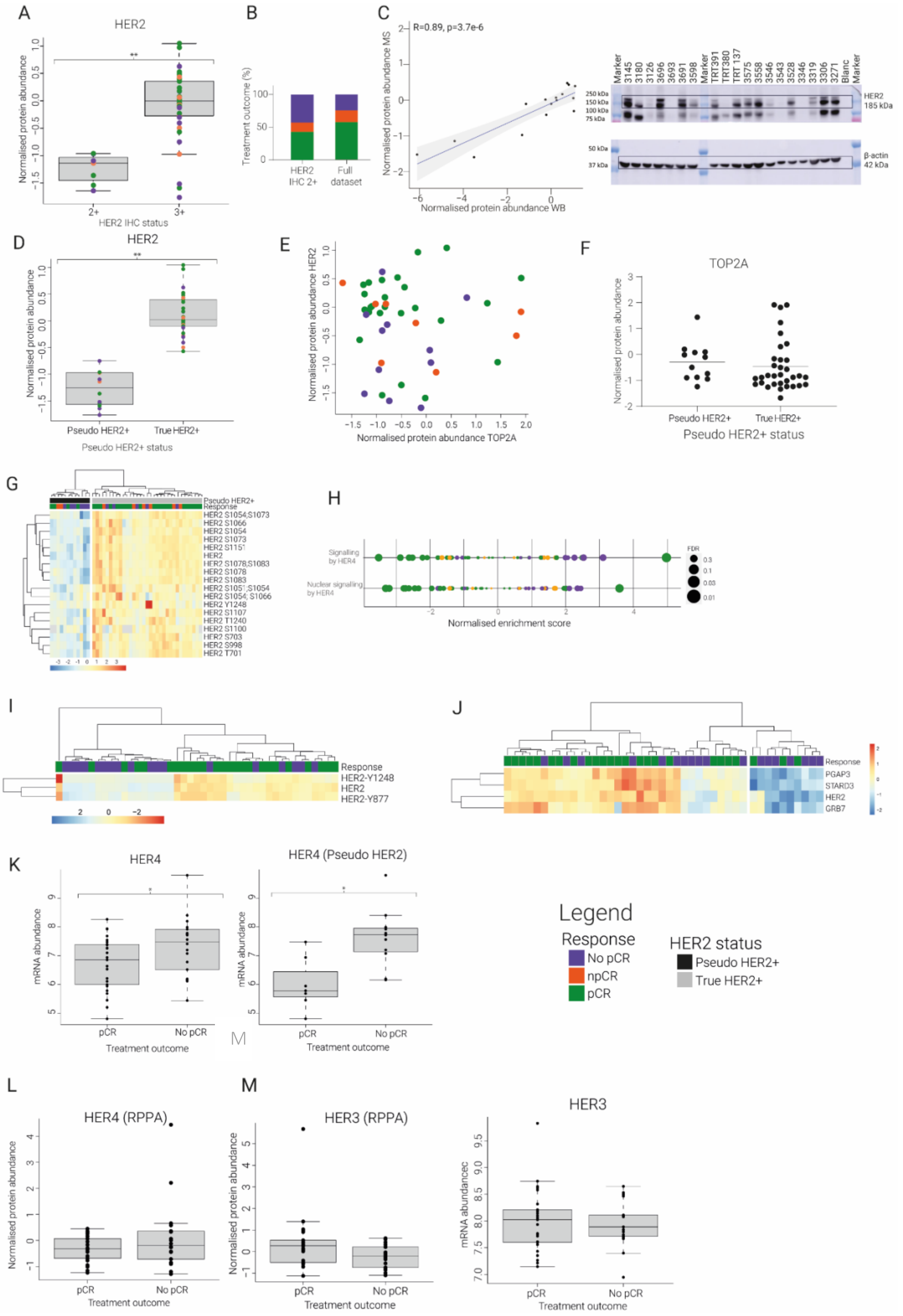

A
HER2
Normalised protein abundance
HER2 IHC status
2+
3+
B
Treatment outcome (%)
HER2 IHC 2+
Full dataset
C
R=0.89, p=3.7e-6
Normalised protein abundance MS
Normalised protein abundance WB
Marker
HER2 185 kDa
β-actin 42 kDa
D
HER2
Normalised protein abundance
Pseudo HER2+
True HER2+
Pseudo HER2+ status
E
Normalised protein abundance HER2
Normalised protein abundance TOP2A
F
TOP2A
Normalised protein abundance
Pseudo HER2+
True HER2+
Pseudo HER2+ status
G
Pseudo HER2+
Response
HER2 S1054;S1073
HER2 S1066
HER2 S1054
HER2 S1073
HER2 S1151
HER2
HER2 S1078;S1083
HER2 S1078
HER2 S1083
HER2 S1051;S1054
HER2 S1054; S1066
HER2 Y1248
HER2 S1107
HER2 T1240
HER2 S1100
HER2 S703
HER2 S998
HER2 T701
H
Signalling by HER4
Nuclear signalling by HER4
FDR
Normalised enrichment score
I
Response
HER2-Y1248
HER2
HER2-Y877
J
Response
PGAP3
STARD3
HER2
GRB7
K
HER4
HER4 (Pseudo HER2)
mRNA abundance
pCR
No pCR
Treatment outcome
L
HER4 (RPPA)
Normalised protein abundance
pCR
No pCR
Treatment outcome
M
HER3 (RPPA)
Normalised protein abundance
pCR
No pCR
Treatment outcome
HER3
mRNA abundancec
pCR
No pCR
Treatment outcome
Legend
Response
No pCR
npCR
pCR
HER2 status
Pseudo HER2+
True HER2+

Supplementary Figure 2. Pseudo HER2 signature is associated with poor treatment response. Related to Figure 1. A) Boxplot of HER2 protein expression shows significantly increased abundance amongst the HER2 IHC 3+ tumours compared to the 2+ tumours. The lowest HER2 expression levels however were found amongst IHC 3+ tumours. B) pCR rate (%) amongst the HER2 IHC2+ tumours (n=7) compared to the full dataset (n=45). C) Correlation plot of HER2 protein expression levels as established by WB compared to MS and the WB raw data. WB HER2 band intensities are normalized to actin loading control. Box indicates quantified bands. D) Boxplot of HER2 protein expression by HER2-status shows significant downregulation of HER2 within the Pseudo HER2+ subgroup; tumours within the Pseudo HER2+ subgroup have the lowest HER2 expression levels. E) Correlation plot between HER2 protein abundance and TOP2A protein abundance coloured by treatment outcome. F) TOP2A protein abundance between Pseudo HER2+ subgroup and true HER2+ subgroup. G) Heatmap of unsupervised clustering of all phosphosites on HER2 shows clustering of all Pseudo HER2+ tumours together. H) Normalised Enrichment Score of PTM-SEA analysis for HER4 signalling. Dot size represents FDR and colour represent patient outcome. I) Heatmap of unsupervised clustering of RPPA HER2 protein and phosphosites shows enrichment of treatment resistant tumours in a cluster with low HER2 and HER2-phosphosite levels. J) Heatmap of unsupervised clustering of PGAP3, STARD3, HER2 and GRB7 mRNA data shows enrichment of treatment resistant tumours with low abundance of all proteins. K) Boxplot of HER4 mRNA levels shows significant upregulation of HER4 mRNA levels in treatment resistant tumours (left). This difference is even bigger within the Pseudo HER2 patient population (right). L) Boxplot of HER4 RRPA data shows no difference in protein abundance between treatment outcome groups. M) Boxplot of HER3 RPPA data (left) and HER3 mRNA data shows no difference between treatment outcome groups. * p-value < 0.05 ** p-value < 0.01

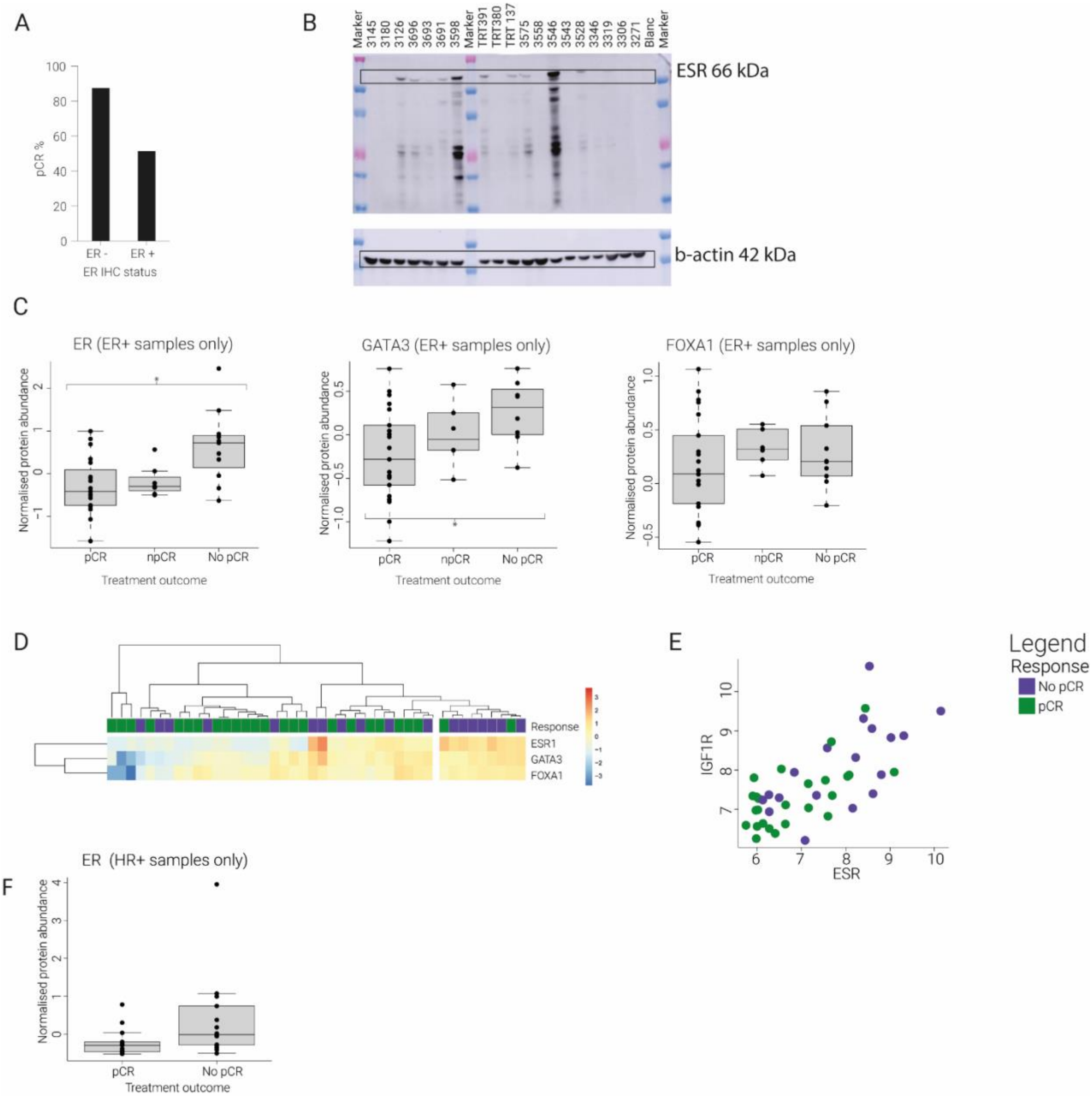

Supplementary Figure 3. ER signalling is associated with poor treatment response. Related to Figure 2. A) pCR rate (%) compared between the tumours with ER- status vs. ER+ as determined by IHC. B) WB data of ESR. Quantified bands indicated by box. ESR abundant was normalized to actin loading control. C) Boxplots of ER, GATA3 and FOXA1 expression amongst the three outcome groups for ER+ tumours only. D) Unsupervised clustering of mRNA expression of ESR, GATA3 and FOXA1 show clustering of treatment resistant patients with the highest ESR, GATA3 and FOXA1 mRNA levels. E) Correlation plot of ESR vs. IGF1R mRNA expression levels, * p-value < 0.05, ** p-value < 0.01. F) Boxplots of RPPA ER expression amongst the two outcome groups for hormone(HR)+ tumours only

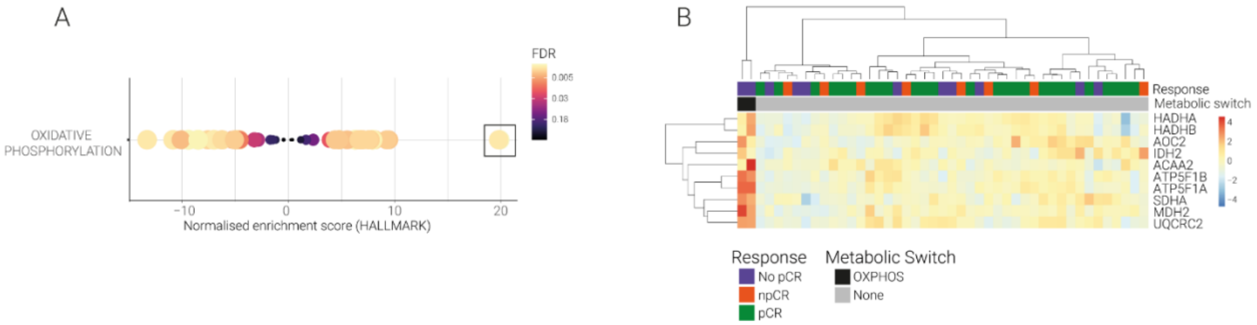

Supplementary Figure 4. Metabolic Switch preconditions tumours for therapy resistance. Related to Figure 3. A) GSEA analysis of oxidative phosphorylation Hallmark signature. Normalised enrichment score (NES) is plotted on the x-axis, dot size and colour represent the FDR. Tumour samples enclosed by black box are samples that have undergone a metabolic switch. B) Heatmap of unsupervised clustering of the top 10 most abundant mitochondrial proteins. Tumour samples that have undergone a metabolic switch are indicated in black. These grouped together and exhibited the highest abundance of mitochondrial proteins.

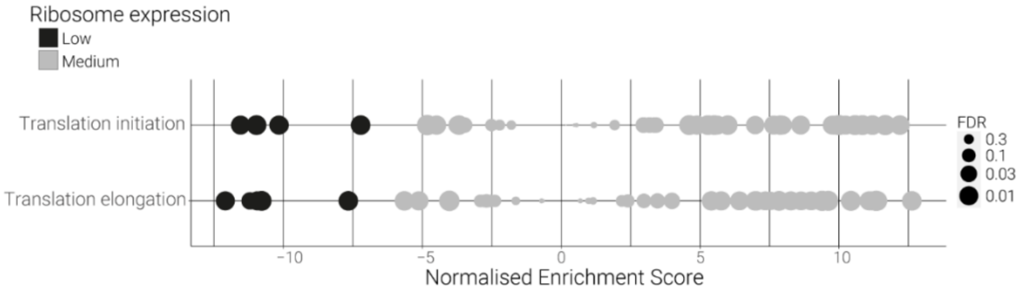

Supplementary Figure 5. UPR-induced cellular dormancy preconditions tumour to resist therapy. Related to Figure 4. Normalised enrichment scores of GSEA analysis of Reactome pathways showed reduced translation initiation and elongation amongst tumours with reduced ribosomal protein levels (shown in black). Dot size represents the GSEA FDR.

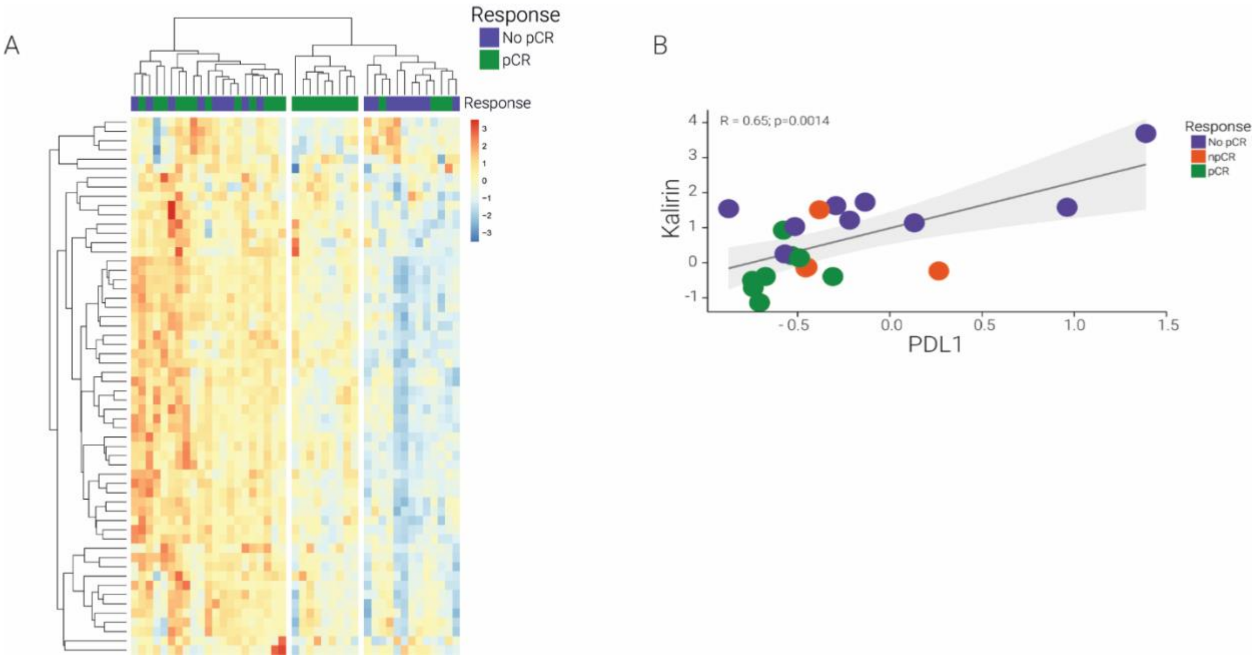

Supplementary Figure 6. Low immune cell infiltration levels correlate with poor treatment response. Related to Figure 5. A) Unsupervised clustering of immune cell marker expression shows clustering of treatment resistant patients with low immune cell marker mRNA levels. B) Kalirin expression correlates with PDL1 levels. Correlation plot comparing protein expression levels, normalized to internal pooled reference and log2 transformed, for all patients in the low-immune subset. Colour code represent patient pCR response. R = person correlation. Grey region represents the 95% confidence interval.
