# Supplementary material for: Deep (phospho)proteomics profiling of pre- treatment needle biopsies identifies signatures of treatment resistance in HER2+ breast cancer
Source: Cell Rep Med. 2023 Oct 3;4(10):101203. doi: 10.1016/j.xcrm.2023.101203 (PMC10591042; doi:10.1016/j.xcrm.2023.101203)
Supplement: Document S2. Article plus supplemental information [file mmc4.pdf]

Cell Reports
Medicine

Article

Deep (phospho)proteomics profiling of pre-treatment needle biopsies identifies signatures of treatment resistance in HER2$^+$ breast cancer

Graphical abstract

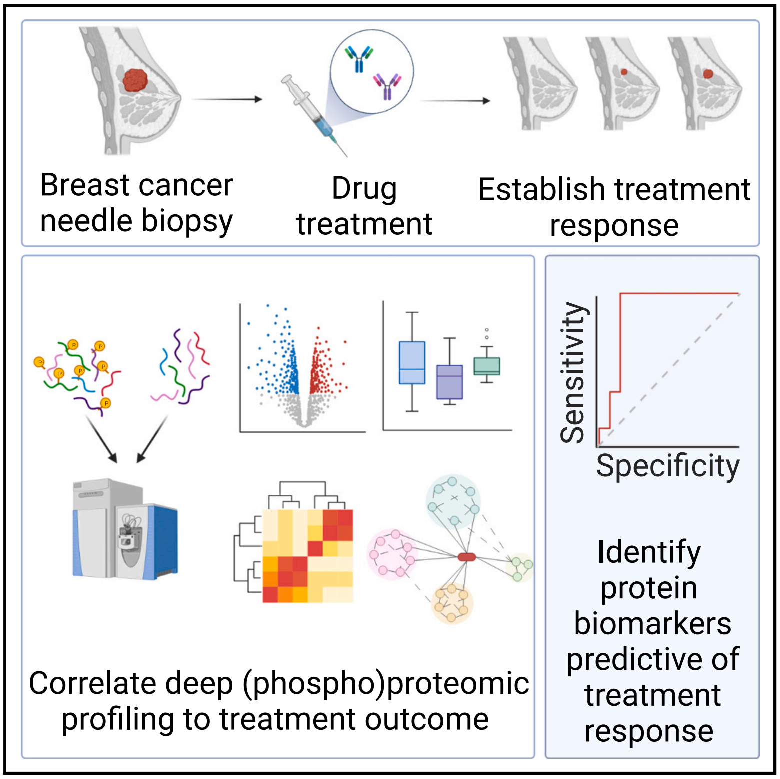

Highlights

- Deep (phospho)proteomics profiling of pre-treatment breast cancer needle biopsies
- Accurate quantification of HER2 and ER abundance improves outcome predictions
- Low tumor infiltration of immune cells is associated with poor treatment response
- Expression levels of HER2, HER4, ER, IGF1R, and Kalirin predict treatment response

Authors

Donna O. Debets, Kelly E. Stecker, Anastasia Piskopou, ..., Gabe S. Sonke, Esther H. Lips, Maarten Altelaar

Correspondence

m.altelaar@uu.nl

In brief

Resistance against targeted therapy in HER2$^+$ breast cancer patients is prevalent. Debets et al. perform (phospho)proteomics profiling of early-stage HER2$^+$ breast cancer biopsies to identify signatures predictive of treatment response. Resistance is multifaceted, and combining receptor protein quantification with corresponding signatures of activation enables better prediction of patient response.

Debets et al., 2023, Cell Reports Medicine *4*, 101203
October 17, 2023 © 2023 The Authors.
https://doi.org/10.1016/j.xcrm.2023.101203

CellPress

Cell Reports Medicine

CellPress
OPEN ACCESS

Article

Deep (phospho)proteomics profiling of pre-treatment needle biopsies identifies signatures of treatment resistance in HER2$^+$ breast cancer

Donna O. Debets,[1,6] Kelly E. Stecker,[1,6] Anastasia Piskopou,[1] Marte C. Liefaard,[2] Jelle Wesseling,[2,3] Gabe S. Sonke,[4,5] Esther H. Lips,[2] and Maarten Altelaar[1,7,*]
[1]Biomolecular Mass Spectrometry and Proteomics, Bijvoet Center for Biomolecular Research and Utrecht Institute for Pharmaceutical Sciences, University of Utrecht, 3584 Utrecht, the Netherlands
[2]Department of Molecular Pathology, The Netherlands Cancer Institute, Amsterdam, the Netherlands
[3]Department of Pathology, Leiden University Medical Center, Leiden, the Netherlands
[4]Department of Medical Oncology, The Netherlands Cancer Institute, Amsterdam, the Netherlands
[5]Department of Medical Oncology, University of Amsterdam, Amsterdam, the Netherlands
[6]These authors contributed equally
[7]Lead contact
*Correspondence: m.altelaar@uu.nl
https://doi.org/10.1016/j.xcrm.2023.101203

SUMMARY

Patients with early-stage HER2-overexpressing breast cancer struggle with treatment resistance in 20%–40% of cases. More information is needed to predict HER2 therapy response and resistance *in vivo*. In this study, we perform (phospho)proteomics analysis of pre-treatment HER2$^+$ needle biopsies of early-stage invasive breast cancer to identify molecular signatures predictive of treatment response to trastuzumab, pertuzumab, and chemotherapy. Our data show that accurate quantification of the estrogen receptor (ER) and HER2 biomarkers, combined with the assessment of associated biological features, has the potential to enable better treatment outcome prediction. In addition, we identify cellular mechanisms that potentially precondition tumors to resist therapy. We find proteins with expression changes that correlate with resistance and constitute to a strong predictive signature for treatment success in our patient cohort. Our results highlight the multifactorial nature of drug resistance *in vivo* and demonstrate the necessity of deep tumor profiling.

INTRODUCTION

Invasive breast cancer (IBC) is a highly heterogeneous disease that relies on subtype classification to prognosticate the disease course and to select treatment strategies.[1] Current IBC patient classification focuses on the expression of three receptor proteins: the estrogen receptor (ER), the progesterone receptor (PR), and the human epidermal growth factor receptor 2 (HER2). Subtype-specific treatment strategies are directed at these receptors and their downstream signaling pathways because these are considered to drive tumor progression. The efficacy of these subtype-driven therapeutic interventions is limited by our ability to further classify IBC within the main subgroups and identify tumors that are truly biologically driven by these targeted receptors.

Approximately 15% of all IBC tumors overexpress HER2.[2] The current standard neo-adjuvant treatment for HER2$^+$ IBC applies targeted therapy using the monoclonal HER2-directed antibodies trastuzumab (TTZ) and pertuzumab (Ptz) in combination with conventional chemotherapy drugs, such as taxanes and carboplatin.[3–6] The introduction of these targeted therapies has improved the clinical outcome for this group of patients considerably, yet treatment resistance, both intrinsic and acquired, occurs because 20%–40% have only partial response to neo-adjuvant treatment.[7] A full understanding of the molecular mechanisms underlying treatment resistance *in vivo* is lacking. Although there has been progress in identifying genomic and transcriptomic features that predict treatment success of HER2-targeted therapies, no biomarkers are currently used in clinic.[8]

The HER2$^+$ subgroup represents a remarkably heterogeneous population of tumors.[9] Poor treatment response in some patients has been attributed to this heterogeneity, because it represents a mismatch between tumor biology and applied therapeutics. For example, HER2$^+$ tumors co-express varying levels of ER, wherein ER expression correlates with treatment outcomes for HER2-targeted therapy.[10] Therefore, enhanced patient stratification could improve clinical outcomes by identifying more suitable therapeutic strategies in some cases and reduction of overtreatment in others. Deep tumor profiling is required to enhance IBC classification and uncover the *in vivo* biological complexity and diversity of treatment resistance in HER2$^+$

This is an open access article under the CC BY license (http://creativecommons.org/licenses/by/4.0/).

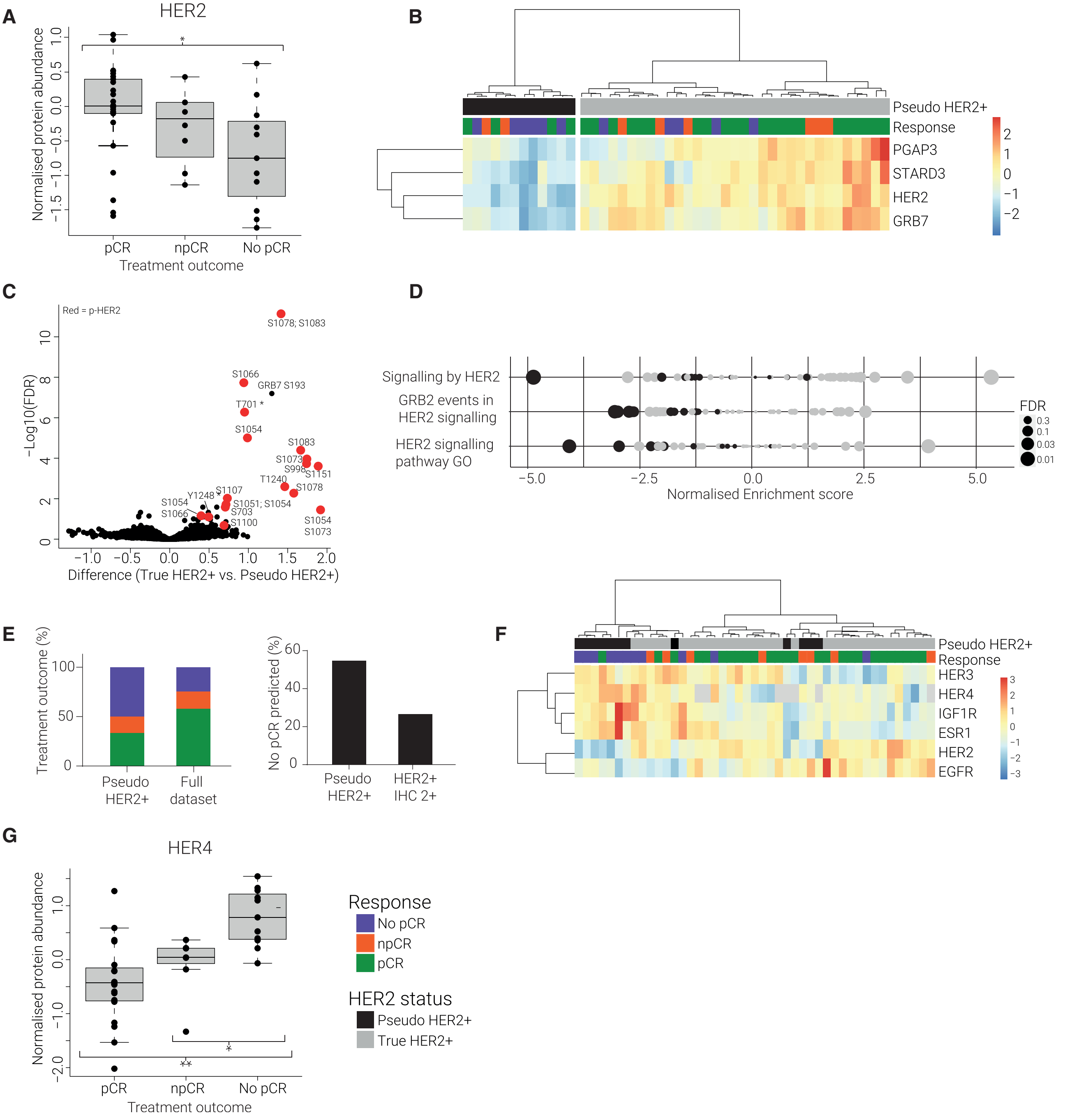

Figure 1. Pseudo-HER2^{+} signature is associated with treatment resistance

(A) Boxplot of HER2 protein expression by treatment outcome group. Protein abundances are normalized to the pooled reference channel. *Indicates p value < 0.05 in unpaired students t test.

(B) Heatmap of unsupervised clustering of HER2 and adjacent genes PGAP3, STARD3, and GRB7 (data are *Z* scored).

(C) Volcano plot comparing phosphosites between true HER2^{+} subgroup vs. pseudo-HER2^{+} subgroup. All phosphosites belonging to the HER2 protein are indicated in red. Difference values are log2 ratios between subgroups.

(D) GSEA of HER2-related Reactome pathways and Gene Ontology. NES (normalized enrichment score) is shown on the x axis; the dot size represents the –log10(FDR). Pseudo-HER2 samples are shown in black.

(E) Left: pCR rate (%) among the pseudo-HER2^{+} subgroup (n = 12) compared with the full dataset (n = 45). Right: number of No pCR samples (%) in pseudo-HER2^{+} subgroup compared with IHC 2+ subgroup.

(legend continued on next page)

tumors. Delineation of the dynamic and complex cellular networks in individual tumor samples opens the door to precision oncology.

In the current study, we perform proteomics and phosphoproteomics profiling of 45 pre-treatment biopsies of patients with early-stage HER2$^+$ IBC (37 ER$^+$ and 8 ER$^-$ cases) to identify molecular signatures predictive of treatment response to neo-adjuvant carboplatin, paclitaxel, TTZ, and Ptz. We demonstrate the feasibility of microscale clinical proteomics; we present deep (phospho)proteomics profiling with high data quality using very limited sample input (half of a 14G needle biopsy). Our data show that IBC subtype classification by accurate quantification of IBC biomarkers combined with the assessment of associated biological features improves treatment outcome prediction. We demonstrate an enhanced IBC classification scheme where signatures of biological activity are used in combination with receptor protein expression data to identify tumors that are HER2 or ER driven. Furthermore, we identify multiple cellular mechanisms that precondition tumors to resist therapy: unfolded protein response (UPR) induced cellular dormancy, a metabolic switch toward oxidative phosphorylation (OXPHOS), and reduced numbers of tumor-infiltrating leukocytes (TILs). Together, the identified resistance mechanisms constitute to a strong signature associated with treatment success in this dataset.

RESULTS

To gain insight into molecular signatures predictive of treatment response for paclitaxel, carboplatin, TTZ, and Ptz (PTC-Ptz), we analyzed 45 treatment-naive early IBC needle biopsies using our microscale (phospho)proteomics workflow (Figure S1). All patient biopsies included in this study were classified as HER2$^+$ with a minimum tumor grade of 2 and tumor cell density of 60% or higher. After needle biopsies were taken, patients completed multiple drug treatment cycles according to the regimen of the TRAIN2 study.[6,11] Finally, treatment response was determined at surgery. The systemic treatment response was categorized as pathological complete response (pCR = ypT0/isypN0) (n = 26), near pCR (npCR) (n = 8), or No pCR (n = 11). If pCR was not achieved, samples were referred to as npCR if <10% of tumor remained and as treatment resistant (No pCR) if >10% of tumor remained. A complete list of patient biopsy details is provided in Table S1.

We obtained deep proteome and phosphoproteome coverage of patient samples using tandem mass tag (TMT) isobaric labeling of digested proteins from limited tissue material (less than half of a 14G needle biopsy, ca. 6–12 mg of fresh frozen tissue). Labeled samples were multiplexed into separate TMT 10-plex sets all sharing an identical pooled reference channel, enabling accurate quantification across TMT sets. Protein and phosphopeptide abundance values were normalized to this shared pooled reference channel within each TMT set. Altogether, 11,088 protein groups and 37,696 phosphopeptides were quantified across the 45 breast cancer biopsies, of which 9,340 protein groups and 11,234 phosphopeptides were identified in at least 75% of the samples (Figures S1B and S1C; Tables S2 and S3). We observed strong longitudinal reproducibility of our peptide and protein quantification demonstrated by the high correlation of the pooled TMT reference channel measurements across TMT sample sets (Figure S1D). The quantitative reproducibility was further determined by replicate samples that were measured across different TMT sets. These replicates showed high correlation in both their proteome and phosphoproteome measurements (Figure S1E) and tight grouping during unsupervised clustering of our datasets (Figure S1F). Finally, we performed unsupervised clustering to confirm that our data did not suffer from any TMT batch effects or clustering bias based on tumor percentages (Figure S1F). Together, these data demonstrate the technical strength and quantitative reproducibility of our microscale (phospho)proteomics workflow.

Enhanced IBC subtype classification improves treatment outcome prediction

Low HER2 activity contributes to treatment resistance

The efficacy of HER2-targeted therapy is partially attributed to the inhibition of HER2 signaling in tumors that are dependent on this pathway for cell growth and proliferation (i.e., HER2-driven tumors).[12] Low activity of HER2 signaling has therefore been proposed as a resistance mechanism against HER2-targeted therapy.[13] Accordingly, we anticipated that identifying tumors that are not HER2 driven would enable detection of poor treatment response in patients.

To identify HER2-driven tumors, we first examined HER2 quantification within the patient biopsies. Current HER2 classification is based on immunohistochemistry (IHC) scoring supplemented by *in situ* hybridization if IHC provides an ambiguous score (2+). Tumors with a 2+ or 3+ score are eligible for HER2-targeted therapy. We compared our proteomics measurements with this classification and found that the IHC scores correlated with HER2 protein levels in general but were inconsistent for some tumors (Figure S2A). We found that a 2+ IHC score could not sufficiently predict treatment response (Figure S2B), whereas protein quantification of HER2 by proteomics measurement showed a strong correlation with pCR status (Figure 1A). HER2 was significantly lower among the No pCR samples in our proteomic data ($p < 0.05$) (Figure 1A). To validate our mass spectrometry (MS)-based measurements of HER2 abundance, we performed western blot analysis on a subset of patient samples and found a strong correlation ($R = 0.89$, $p = 3.7e{-6}$) in relative protein expression levels (Figure S2C). Together, these data indicate that a more quantitative readout for HER2 protein levels, compared with current IHC semi-quantitative classification, may better discern between treatment outcome groups.

To support the HER2 protein levels measured in our dataset, we evaluated additional features that indicate HER2 activity. Herein, we identified a subset of samples, referred to as

(F) Heatmap of unsupervised clustering of HER2 family members (EGFR, HER3, HER4), ER, and IGF1R expression levels groups the samples into two main clusters. Left cluster shows enrichment of pseudo-HER2$^+$ patients with No pCR and high levels of HER3, HER4, IGF1R, and ER. (data are *Z* scored).
(G) Boxplot of HER4 protein expression by treatment outcome group (FDR = 0.02). *$p < 0.05$; **$p < 0.01$.

"pseudo-HER2+," which do not appear HER2 driven despite being eligible for HER2-targeted therapy. First, we evaluated the expression of genes adjacent to HER2 in the genome (PGAP3, STARD3, and GRB7) as a proxy for regional genome activity. Pseudo-HER2 samples showed low protein expression of these *cis* genes, indicating reduced genome activity and consequently low HER2 expression, even in patients possessing a high number of HER2 genome insertions (Figures 1B and S2D). Expression levels of TOP2A, however, did not correlate to HER2 expression levels and were similar for the pseudo-HER2+ subgroup (Figures S2E and S2F). Second, we observed that the pseudo-HER2+ subgroup displayed a lower activation state of HER2 as demonstrated by significant downregulation of HER2 phosphorylation sites (false discovery rate [FDR] < 0.05) (Figures 1C and S2G). Finally, we found that HER2 downstream signaling was decreased within the pseudo-HER2+ tumors (Figure 1D). We next assessed the pCR status of the patients in our pseudo-HER2+ group and found that pseudo-HER2+ classification was a far stronger predictor for treatment resistance compared with the HER2 IHC score (Figure 1E). These data indicate that patient stratification based on a multi-level assessment of HER2 features improves predictions for patient response to treatment.

We next set out to explore the differences between the pCR and No pCR patients within the pseudo-HER2+ subgroup. We found that pseudo-HER2+ tumors with high expression levels of HER2 family members (HER3 and HER4) and alternative hormone receptors (ER and insulin growth factor 1 receptor [IGF1R]) were generally treatment resistant (Figure 1F). This finding suggests that these receptors could provide a compensating mechanism, bypassing HER2 dependency in No pCR patients within the pseudo-HER2+ subgroup. For HER3, this is in contrast with previous research, in which low HER3 levels were linked to treatment resistance.[14] Notably, HER4 was also found to be significantly upregulated (FDR = 0.02) among the treatment-resistant tumors within the full dataset (regardless of the pseudo-HER2+ status) (Figure 1G). This was accompanied by increased downstream HER4 signaling (Figure S2H) and suggests a more widespread role of HER4 in treatment resistance.

To validate our findings in a larger BC clinical dataset, we analyzed biopsy data from the I-SPY2 neoadjuvant trial (NIH identifier: NCT01042379), which contains pre-treatment biopsies matched to patient outcomes for 10 different treatment arms.[15] We extracted reverse-phase protein array (RPPA) and mRNA data for 43 HER2+ patients who received paclitaxel + pertuzumab + trastuzumab (PPT) treatment, of which 25 patients achieved pCR. We first examined HER2 RPPA protein expression levels and found that, in agreement with our observations, HER2 abundance and HER2 phosphorylation sites were significantly lower in No pCR patients compared with pCR patients (Figure S2I). We next evaluated the "pseudo-HER2" signature in patient mRNA data and found that biopsies possessing low expression of HER2 adjacent genes (PGAP3, STARD3 GRB7) were enriched for No pCR treatment outcomes (Figure S2J), supporting our proteomics findings. Finally, we examined the expression of HER3 and HER4 family members and found significant mRNA upregulation of HER4 in No pCR patients (Figure S2K). HER4 upregulation was even more pronounced when we evaluated treatment response within the "pseudo-HER2" patient subset (Figure S2K). Interestingly, we did not find significant HER4 upregulation in RPPA protein data. We also did not observe any HER3 upregulation in No pCR patients in mRNA or RPPA measurements (Figure S2M).

High ER signaling is associated with treatment resistance, especially in combination with high IGF1R activity

In addition to the determination of the HER2 status, IBC tumors are also classified based on IHC staining of ER. A negative ER IHC status has been associated with better treatment response.[10] Accordingly, we found a higher pCR rate among ER tumors in our dataset (Figure S3A). Positive ER classification by IHC scoring, however, did not show an enrichment of treatment-resistant patients (Figure 2A). ER− tumors are likely responsive to treatment, whereas the ER+ population has a heterogeneous treatment outcome. We found that ER protein expression levels measured by MS were generally associated with the IHC status (FDR < 0.05). However, there was a large overlap in ER protein expression levels between the ER− and ER+ tumors (Figure 2B). To validate our MS quantification, we performed anti-ER WB analysis on a subset of patient biopsies and found a strong correlation in our protein quantitation ($R = 0.86$, $p = 1.5e-5$) (Figures 2C and S3B). Together this demonstrates that even though a negative ER IHC status is generally predictive of pCR, ER+ tumors have a mixed treatment outcome and thus treatment-resistant tumors cannot be detected by this classification. Furthermore, for some tumors the ER protein expression levels did not reflect the IHC classification. These observations indicate that further discrimination between ER+ patients is needed to better identify non-responsive patients.

We hypothesized that among the ER+ tumors a subset of samples was truly ER dependent, resulting in a poor treatment response. To establish whether ER-dependent signaling could be discerned within the ER+ tumors, we first analyzed the protein expression levels of ER and ER-associated proteins GATA3 and FOXA1. The results revealed that ER and GATA3 protein expression were significantly upregulated among the treatment-resistant tumors within the ER+ subgroup (Figure S3C) ($p = 0.007$ and 0.046, respectively). FOXA1 contrarily did not significantly differ between the outcome groups (Figure S3C). Second, we quantified ER-dependent signatures in the tumor proteome using gene set enrichment analysis (GSEA). Combining the results together identified a subgroup of tumors displaying an ER-dependent signature, characterized by high expression levels of ER, FOXA1, and GATA3 and enrichment of ER-related signaling (Figure 2D). This classification of tumors, based on accurate ER protein quantification by proteomics and ER-driven biological features, was a better predictor of treatment resistance than the ER IHC status (Figure 2A). ER-dependent tumors showed >50% increase in No pCR patient enrichment compared with IHC ER+ tumors.

We next set out to investigate the differences between the pCR and No pCR tumors within the ER-dependent subgroup. We found that the correlation between the ER and IGF1R expression levels was very high and, in agreement with previous findings,[16] that high expression levels of IGF1R and ER were correlated with treatment resistance (Figure 2E).

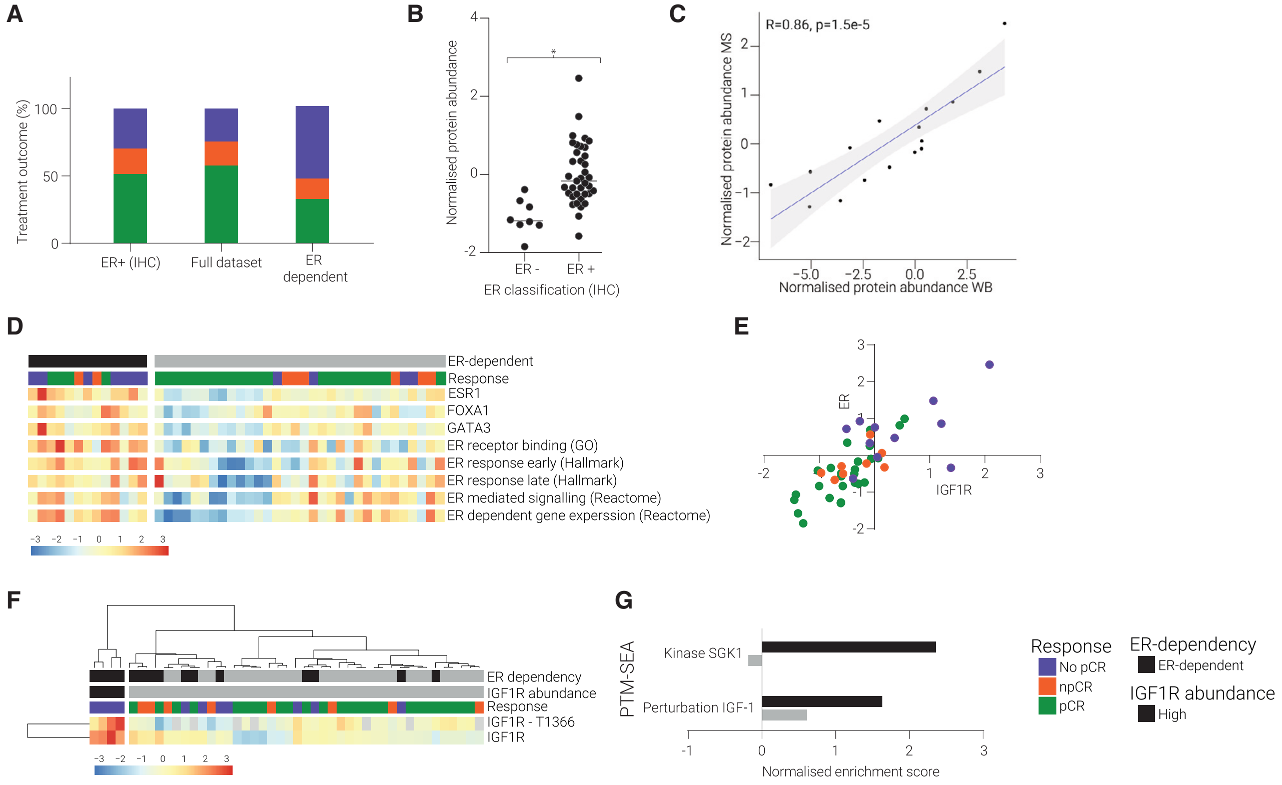

Figure 2. High ER signaling predicts poor treatment response, especially in combination with high IGF1R activity

(A) pCR rate (%) for the ER-dependent subgroup (n = 13), tumors with an ER + IHC status (n = 37), and the full dataset (n = 45).

(B) ER protein expression (determined by proteomics) by ER IHC status. * FDR < 0.05

(C) Correlation plot of ESR protein abundance as established by western blotting (WB) compared with MS.

(D) Heatmap showing increased ER signaling and increased levels of FOXA1 and GATA3 among a subgroup of tumors that were classified as ER dependent. (data are *Z* scored).

(E) Correlation plot of the ER and IGF1R expression levels. Point color represents the treatment outcome. Pearson correlation: 0.78.

(F) Heatmap of unsupervised clustering of IGF1R and phospho-IGF1R-T1366 groups four patients together with the highest expression levels. These tumors were resistant to treatment and ER dependent. (data are *Z* scored).

(G) PTM-SEA analysis NESs averaged for the patients with a high IGF1R expression (in black) compared with the rest of the data. An increased enrichment score was found for SGK1 kinase activity and IGF1 perturbation among the tumors expressing high levels of IGF1R.

Unsupervised clustering identified a subset of four tumors that were ER dependent, treatment resistant, and exhibited the highest IGF1R and phospho-IGF1R levels (Figure 2F). These elevated (phospho-)IGF1R expression levels were linked to increased IGF1R downstream signaling, as demonstrated by increased phosphorylation activity caused by IGF1 (an important IGF1R ligand[17]) and high SGK1 activity (a kinase downstream of IGF1R) (Figure 2G). Combined, these data suggest that increased IGF1R activity within the ER-dependent subgroup may contribute to treatment resistance. This is in line with previous research that has suggested interplay between ER and IGF1R[18,19] and a correlation with treatment resistance.[16]

Our data revealed that patient stratification based on the quantitative assessment of ER by proteomics, combined with the analysis of ER-associated proteins (GATA3 and FOXA1) and ER-related signaling, identified a subset of tumors with an ER-driven signature. This signature was a better predictor for treatment resistance than ER IHC scoring in this dataset. Tumors with an ER-dependent signature and high IGF1R activity had an especially poor treatment response. To validate these findings in an independent patient cohort, we analyzed ER protein expression and ER, IGFR1, FOXA1, and GATA2 mRNA expression levels in the I-SPY2 neoadjuvant dataset.[15] In alignment with our MS results, we found that patient tumors possessing high expression of ER, FOXA1, and GATA2 mRNA showed enrichment in No pCR response (Figure S3D). We also saw a strong correlation between ER and IGFR1 mRNA expression levels (Figure S3E). Lastly, we found that within hormone-positive tumors, ER protein levels were higher in No pCR patients than pCR patients (Figure S3F).

Treatment-resistant tumors are preconditioned to evade therapy

Next, we performed an exhaustive analysis to identify further associations between molecular signatures and patient pCR status. We focused on those proteins showing statical significant

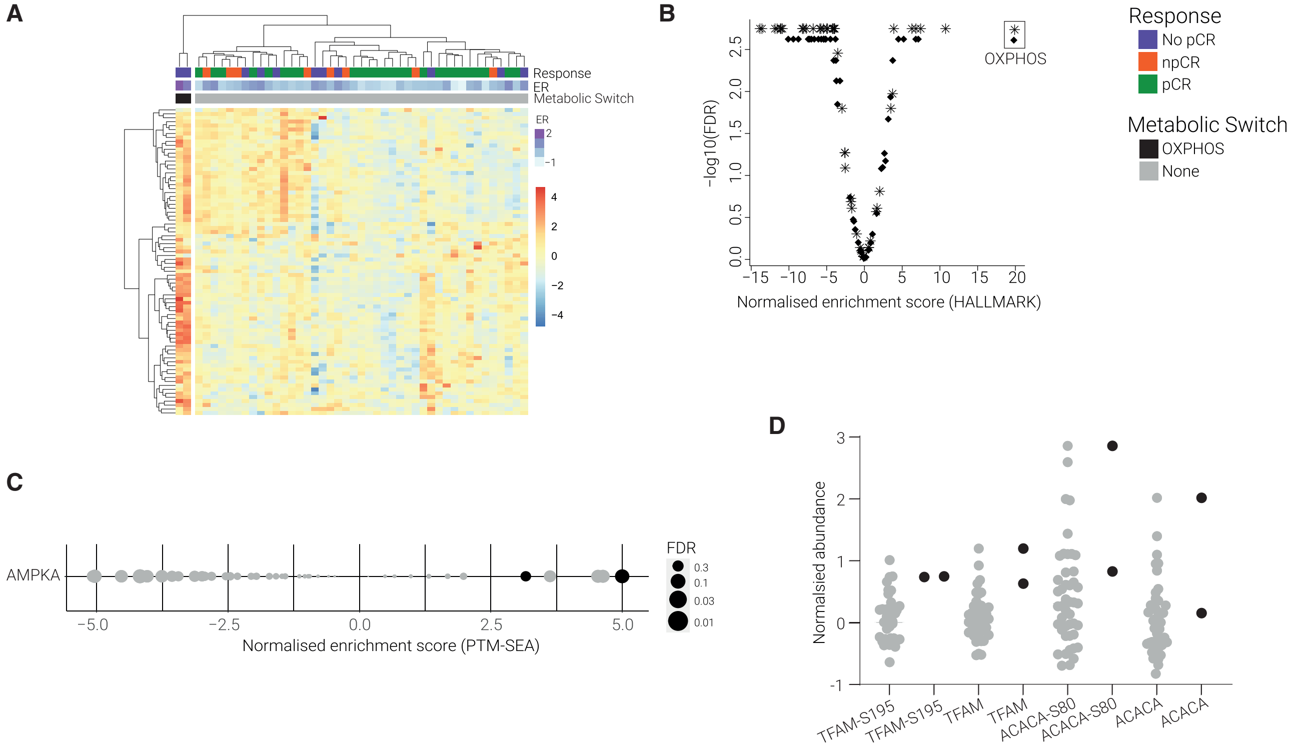

Figure 3. Increased OXPHOS activity preconditions tumors to resist therapy

(A) Heatmap of unsupervised clustering of proteins involved in OXPHOS Reactome pathway grouped two patients together. They were treatment resistant and showed clear increase in OXPHOS proteins. (data are *Z* scored).

(B) Volcano plot of GSEA of hallmark signatures of the two tumors marked with increased levels of OXPHOS. NESs plotted against the –log10(FDR). Hallmark term OXPHOS indicated in the box.

(C) PTM-SEA analysis of AMPKA kinase activity. NESs are plotted on the x axis, dot size represents the FDR score, and color represents the metabolic switch.

(D) Dotplot showing the log2 normalized phosphosite and protein abundance. Dot color represents the metabolic switch.

difference within the patient samples for further bioinformatics analysis and literature mining. This resulted in the observation of several important biological processes observed in subsets of the patient samples that could be involved in therapy resistance.

Increased OXPHOS activity preconditions tumors to resist therapy

To determine whether treatment-resistant tumors are metabolically preconditioned to evade drug therapy, either targeted treatment or chemotherapy because these individual responses cannot be distinguished in our dataset, we looked for upregulation of metabolic pathways known to be associated with treatment resistance. Our data revealed a distinct upregulation of proteins involved in OXPHOS among two treatment-resistant tumors (Figure 3A). GSEA showed a stark enrichment of OXPHOS activity among these same tumor samples (Figure 3B), but not in any of the other tumors (Figure S4A). OXPHOS metabolic reprogramming has been recognized as an emerging hallmark of cancer and is associated with acquired drug resistance.[20–22] Although our observation is in a very small number of patients, the upregulation of the OXPHOS metabolic profile is exceptionally strong and unambiguous.

The OXPHOS signature was accompanied by the highest expression levels of ER (Figure 3A) and increased AMP-activated protein kinase (AMPK) activity (Figure 3C). This agrees with previous research showing that OXPHOS can be activated by ER-mediated activation of AMPK in response to glucose deprivation.[23] We also find increased phosphorylation of known AMPK targets Transcription Factor A (TFAM) and Acetyl-CoA carboxylase 1 (ACACA) (Figure 3D). AMPK-mediated activation of TFAM and ACACA has been shown to stimulate OXPHOS and mitochondrial biogenesis *in vitro* and *in vivo.* 22[24,25] The increased expression levels of mitochondrial proteins found in the two samples with high OXPHOS activity could be the result of increased mitochondrial biogenesis (Figure S4B). Together these data suggest that ER-mediated activation of AMPK may be responsible for the increased OXPHOS found in these tumors.

UPR-induced cellular dormancy preconditions tumors to resist therapy

A significant decrease of the entire ribosomal machinery was found among five tumor samples, of which four were treatment resistant (Figure 4A). These tumors showed decreased expression levels of proteins involved in translation, indicative of a dormant cell type (Figures 4B and S5). We hypothesized that this non-proliferative cell type may prove less responsive to cell-cycle-dependent chemotherapy, resulting in treatment resistance.

Among the tumors with this dormant cell type, we found increased levels of Stanniocalcin-2 (STC2) (Figures 4B and 4C)

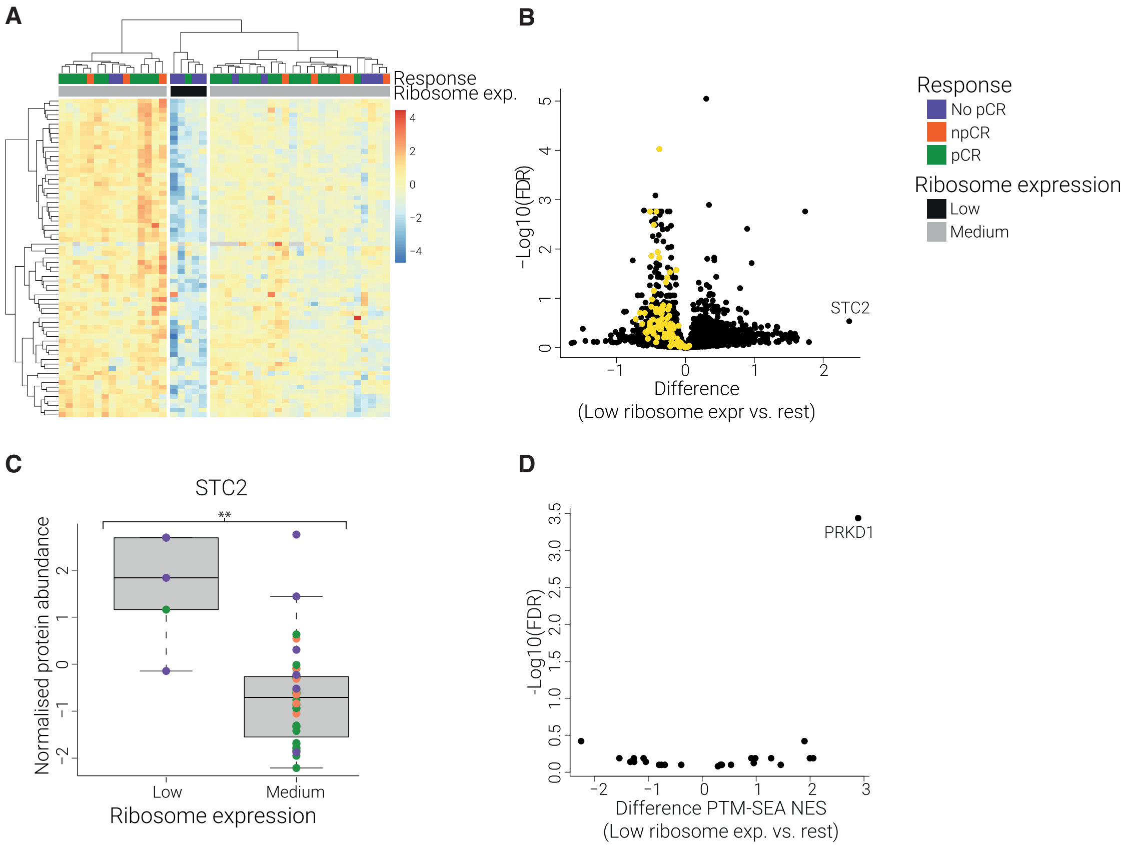

Figure 4. UPR-induced cellular dormancy preconditions tumors to resist therapy

(A) Heatmap of unsupervised clustering of all ribosomal proteins grouped five patients together with the lowest abundance of these proteins. Four of these were resistant to therapy. (data are *Z* scored).

(B) A t test was performed on the proteins within the dataset comparing the five tumor samples with the low-ribosomal proteins against the rest. Difference is plotted on the x axis, and −log10(FDR) on the y axis. Proteins in yellow are involved in translation initiation (Reactome pathway).

(C) Boxplot of STC2 comparing the tumors expressing low amounts of ribosomal proteins vs. the rest. **p < 0.05. Dot color represents the treatment outcome.

(D) A t test was performed on PTM-SEA kinases NESs, including all kinases that were found enriched (FDR < 0.05) in at least one of the low-ribosomal tumors. PDK1 was clearly upregulated among the low-ribosomal tumor samples.

($p < 0.05$). Previous research has shown that a rapid upregulation of STC2 is associated with the UPR.[26,27] UPR is triggered by the accumulation of misfolded proteins within the endoplasmic reticulum and aims to reinstate cellular homeostasis by clearance of the endoplasmic reticulum and constriction of protein synthesis. UPR-induced cellular dormancy has been associated with chemotherapy resistance.[28] The upregulation of STC2 among the dormant tumors suggests that UPR may be activated and responsible for the non-proliferative cell type.

We also found increased PDK1 activity among the dormant tumors (Figure 4D). Upregulation of PDK1 has been shown to circumvent endoplasmic reticulum stress-induced apoptosis in tumor cells.[29] Hence the increased PDK1 activity found in these tumors could provide a pro-survival mechanism upon endoplasmic reticulum stress.

These data indicate that increased UPR could result in a dormant cell type in a subset of tumors that were largely treatment resistant. UPR and endoplasmic reticulum stress have been linked to acquired therapy resistance against many chemotherapeutics.[30–32] Our data suggest that these processes are not merely secondary resistance mechanisms but can also play a role in *de novo* treatment resistance.

Low immune cell infiltration in the TME indicates poor treatment response

Evasion of immune system clearance is a prerequisite for successful tumor progression, and excluding lymphocytes from the tumor microenvironment is one mechanism that enables immune escape. Accordingly, we anticipated that patients who respond poorly to therapy possess low levels of immune cell infiltration in their tumor biopsies. Using a validated selection of immune cell markers,[33] we found that >80% of No pCR tumors had depleted levels of immune cells (Figures 5A and 5B). Furthermore, we found that this "low immune infiltration" patient cohort showed strong downregulation of immune-related signatures within their proteome, whereas the opposite was true for "high immune infiltration" patients (Figure 5C). This demonstrates that both a curated list of specific cell markers and a global analysis of proteome signatures identify the vast majority of non-responders as being immune depleted. We next validated this observation in the I-SPY2 neoadjuvant dataset by clustering patient mRNA data based on the

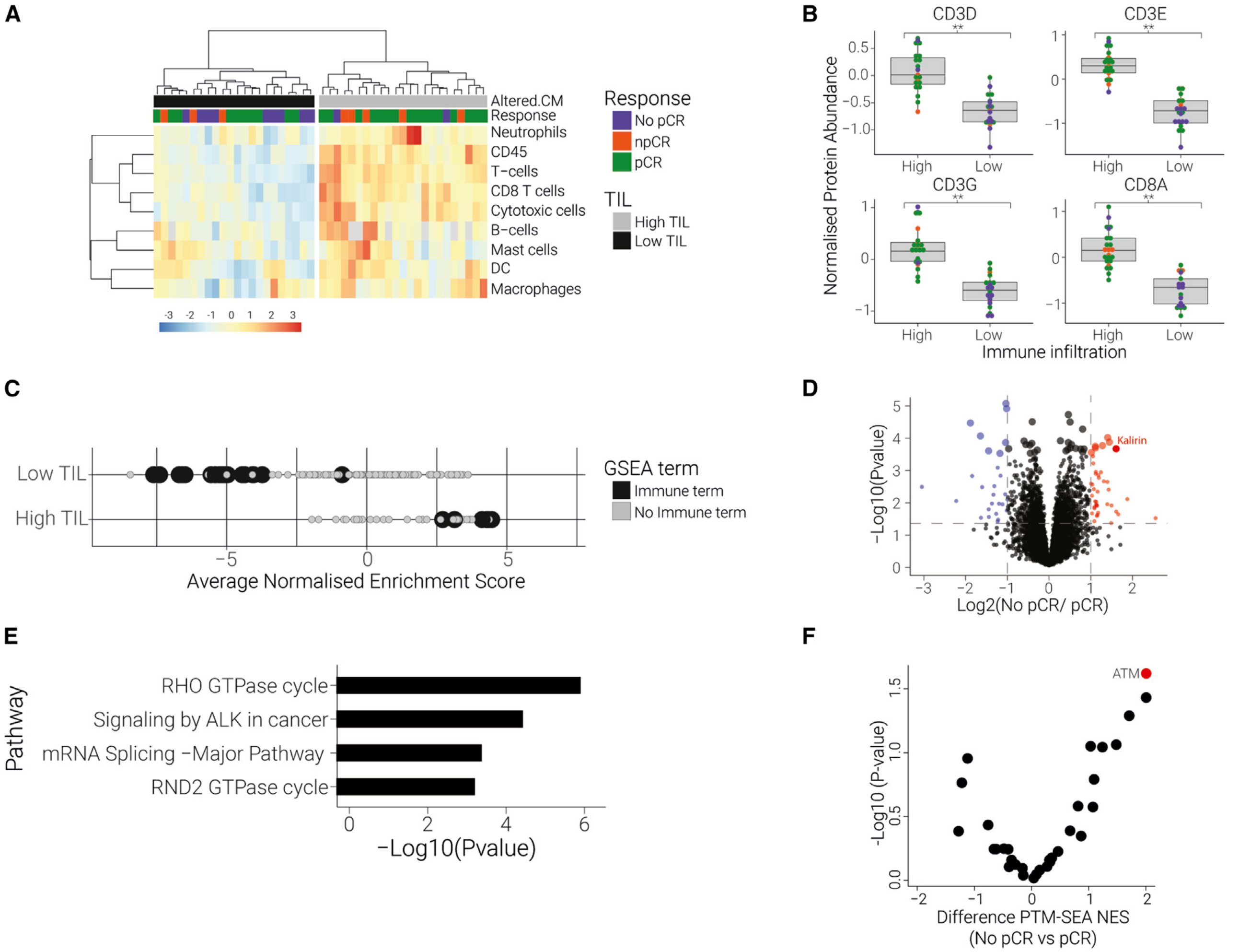

Figure 5. Low immune cell infiltration and high KALRN levels correlate with poor treatment response

(A) Unsupervised clustering of validated leukocyte markers shows a distinct grouping of tumors that contain reduced immune cells (Low TIL); this group includes nine No pCR patients. Data represent scaled abundances.

(B) Downregulation of T cell marker proteins in the low TIL patient subset. Boxplot of T cell protein expression in all samples, grouped by clusters defined in (A). $^{**}p < 5e{-}9$. Dot color represents the treatment outcome.

(C) Downregulation of immune-related pathways in low-TIL sample subset. Dots represent individual GSEA pathways that were significantly enriched (FDR < 0.05) in at least 75% of the samples in each group. Black dots represent immune-related GSEA terms; x axis represents average NES for each group.

(D) Upregulation of Kalirin in No pCR tumors within the low-TIL patient group. Volcano plot comparing protein abundance between low-TIL vs. high-TIL subgroup. Vertical bars indicate 2-fold change in protein abundance. Blue and red dots denote significantly downregulated or upregulated proteins, respectively. Larger dot size represents FDR < 0.1.

(E) Differentially regulated phosphosites in No pCR tumors within the low-TIL group show enrichment of Rho GTPase pathway (FDR < 0.001).

(F) ATM kinase activity is enriched in No pCR tumors within the low-TIL group. A t test was performed on PTM-SEA kinases NESs, including all kinases that were found enriched (FDR < 0.05) in at least three patients.

Danaher et al.[33] immune cell marker panel. Here we found that the cluster of patients showing low levels of TILs was composed of 70% No pCR patients (Figure S6A).

The immune-depleted tumor subset we identified in our dataset was not exclusive to No pCR patients. Therefore, we next examined which features within the low-immune group were enriched specifically in treatment-resistant patients. We identified the guanine nucleotide exchange factor Kalirin as the one of the most upregulated proteins in the No pCR patient subset (>3-fold increase, $p < 0.0003$, FDR < 0.1) (Figure 5D). Kalirin is known to regulate specific Rho GTPases and plays a vital role in neuronal plasticity.[34] Interestingly, loss-of-function mutations in Kalirin were recently identified as a biomarker for positive immunotherapy response across 10 different cancer types.[35] Functional Kalirin protected tumors from DNA damage through activated DNA-damage-repair mechanisms mediated by Rho GTPases, thus contributing to reduced neoantigens and poor immune clearance upon reactivation of the immune system.[35] Accordingly, we found Rho GTPase signaling as the most differentially regulated pathway between No pCR and pCR samples

CellPress
OPEN ACCESS

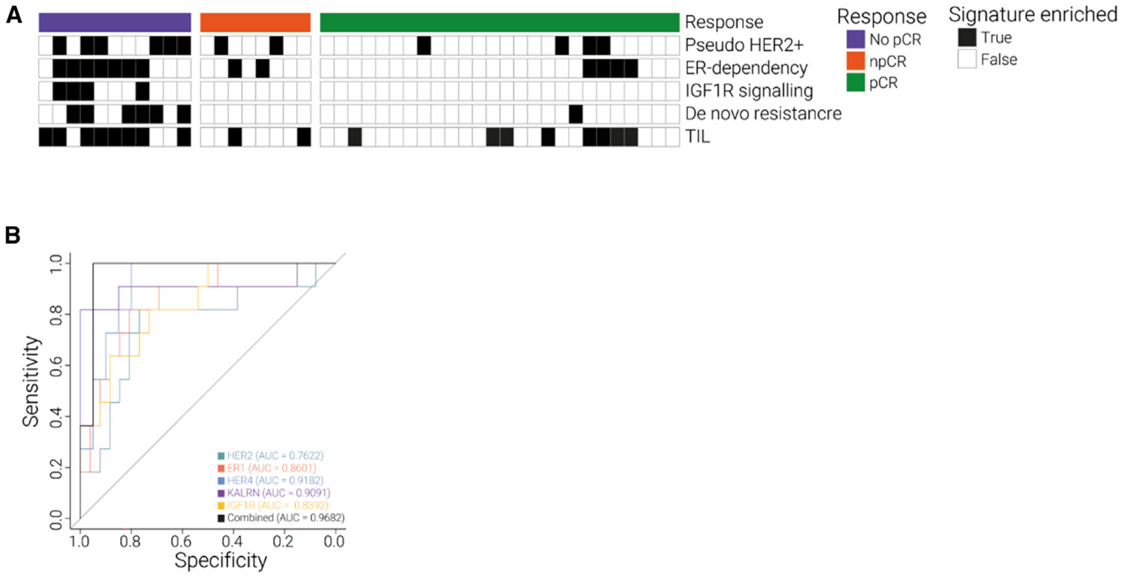

Figure 6. Multifaceted nature of treatment resistance *in vivo*

(A) Overlap of resistance mechanisms was found among treatment-resistant tumors.

(B) ROC curve of proteins representative of the main resistance mechanisms shows great sensitivity and specificity.

within the low-immune subset in our phosphoproteomics data (Figure 5E). We also identified ATM kinase, a central activator of DNA damage response, as significantly activated in No pCR patients compared with pCR patients in this subset (Figure 5F). In contrast with previous findings, however, we identified a positive correlation between Kalirin and PD-L1 protein expression levels (p = 0.0014) in tumor biopsies[35] (Figure S6B). Together, our data demonstrate that non-responsive patients in the low-immune patient cohort have uniquely elevated levels of Kalirin and increased activation of associated downstream pathways. This finding suggests that higher Kalirin levels may participate in protecting tumors from immune cell clearance in No pCR patients, even after targeted therapy.

Treatment resistance is multifaceted, and the combined resistance mechanisms are strongly associated with treatment success

We aimed to assess whether the resistance mechanisms we identified constitute a protein signature associated with treatment outcome. We found that multiple mechanisms coincide in any given treatment-resistant tumor (Figure 6A). Thus, rather than the alteration of a single protein or pathway, the combination of drug-evading mechanisms drives tumor fate. This highlights the multifaceted nature of drug resistance *in vivo* and illustrates the need to take a combination of these features into account to enable a more accurate prediction of treatment outcome. For example, a subset of treatment-resistant patients had both a pseudo-HER2$^+$ signature and showed ESR dependency. This group of patients might benefit from ESR-directed therapy rather than HER2-targeted therapy.

To evaluate the proteome signatures we identified, we selected five proteins representative of the most prevailing resistance mechanisms: HER2, HER4, ER, IGF1R, and Kalirin. Accurate quantification of this panel of proteins by proteomics analysis was strongly associated with treatment response with high sensitivity and specificity, yielding a receiver operating characteristic (ROC) score of >0.95 (Figure 6B). The improved area under the curve (AUC) for a combination of resistance mechanisms compared with the individual proteins (Figure 6B) highlights the complex nature of therapy resistance *in vivo* and underscores the need for deep tumor profiling by system-wide quantitative analysis.

DISCUSSION

In this study, we performed deep proteomics and phosphoproteomics profiling of 45 HER2$^+$ breast cancer tumors prior to the start of neo-adjuvant treatment with PTC-Ptz to identify molecular signatures predictive of treatment response. We show that enhanced IBC subtype classification, based on proteomics quantification of IBC biomarkers (HER2 and ER) combined with the assessment of associated biological features (such as phosphorylation abundance, expression levels of related proteins and activity of downstream signaling), improves treatment outcome prediction. We demonstrate that these observations are conserved in a second patient cohort of HER2$^+$ pre-treatment biopsies. Furthermore, we identify cellular mechanisms that precondition tumors to evade drug treatment: UPR-induced cellular dormancy, a metabolic switch toward OXPHOS and low levels of immune cell infiltration. These resistance mechanisms combined constitute a strong signature associated with treatment success within our patient cohort. Our study highlights the multifactorial nature of drug response *in vivo* and demonstrates the necessity of deep tumor profiling.

We find that the current tumor classification of HER2 and ER can be improved by quantification of these IBC biomarkers

combined with assessment of associated biological features. By categorizing patients as pseudo-HER2 or ER-driven using this combined approach, we were able to identify an enriched population of patients who were resistant to treatment. In contrast, we did not observe any predictive value in the expression of PR in IHC scores or proteome data (data not shown). This is in line with common clinical standards in which PR scoring is not utilized to drive treatment decisions.[36] We validated our pseudo-HER2 and ER-driven findings in an independent cohort of neoadjuvant samples with matched treatment regimens in the I-SPY2 dataset. Extended analysis of the entire HER2$^+$ patient group within this large clinical trial further supports our observations that no pCR outcomes for patients are associated with low HER2$^+$ protein expression and low HER2$^+$ activity.[37] The concept that better patient stratification can be achieved through enhanced IBC biomarker assessment is also in line with research that demonstrates the highly heterogeneous nature of tumors classified as HER2$^+$ by IHC/fluorescence *in situ* hybridization (FISH)[9,14] and research showing the subjective nature of ER classification by IHC scoring.[38] It is important to note that tumor heterogeneity can contribute to discrepancies between measurements and cannot be ruled out as a factor influencing IHC and proteome differences in this study. Nevertheless, the shortcomings in the existing classification procedures hamper the effective deployment of subtype-specific therapeutic interventions. Improvements in patient stratification could directly affect clinical decision-making because suitable treatment strategies for the different IBC subtypes are readily available.

Although our data and the data from Clark et al.[37] indicate that improved treatment precision can be achieved through combining protein activation data with protein abundance measurements in neoadjuvant biopsies, it is still challenging to sufficiently stratify patients to a degree that warrants clinical implementation. In the current dataset, 30% of patients within the "ER-dependent" classification achieved pCR, indicating the applied treatment regimen was appropriate. To achieve stronger separation between pCR and No pCR patients using pre-treatment biomarkers, more *in vivo* data are needed to identify molecular signatures that correlate with patient response. Furthermore, quantification of relevant biomarkers must be translated from relative to absolute abundances for clinical implementation. This can be achieved by using multiplexed targeted proteomic analysis with spiked-in standard for absolute quantification.

Our data suggest that overexpression of HER4 could compensate for HER2 inhibition, contributing to therapy resistance. In support of our findings, we observed a similar increase in HER4 expression in pre-treatment biopsies from patients who did not respond to PTT therapy in the I-SPY2 clinical trial. Nevertheless, the role of HER4 in breast cancer remains ambiguous; increased HER4 levels have been linked to a favorable disease course, yet have also been associated with poor outcomes and trastuzumab resistance.[14,39–41] Furthermore, our findings are inconsistent with previous findings in which low HER2, HER3, and HER4 levels were linked to treatment resistance against trastuzumab and pertuzumab.[14] This suggests that the predictive value of HER4 expression is highly context dependent and requires further investigation.

Our results also indicate a potential role of IGF1R signaling in therapy resistance. This finding is in agreement with previous research and supports efforts into the development of IGF1R inhibitors.[16] The clinical benefits of these inhibitors have been very limited, however, likely because of the lack of patient stratification prior to study inclusion.[42] Our data clearly indicate that only a subset of treatment-resistant tumors could benefit from IGF1R-targeted therapy (Figures 2D and 2E). Furthermore, the efficacy of IGF1R inhibition is context dependent, as shown by the potential interplay between the ER and IGF1R.[18,19] This again highlights the need for precision oncology by system-wide molecular profiling to identify these therapeutic vulnerabilities and put them in their biological context on an individual patient basis.

Another factor we identified to be associated with poor treatment response in our dataset is low levels of immune cell infiltration in the tumor biopsies. Consistent with previous findings in triple-negative BC patients,[43] where pCR rates correlated with TIL abundance, we found that No pCR patients were significantly enriched in the low-TIL patient subset. We validated our findings in the I-SPY2 patient cohort, where we saw similar enrichment of no PCR patients within the low-TIL patient subset. Interestingly, within our low-TIL subset, we observed a significant increase in Kalirin protein expression and subsequent enrichment of Kalirin-associated signaling. Mutations in the KALRN gene have been identified as a biomarker for positive immunotherapy response, which is attributed to the loss of Kalirin function in activating DNA-damage repair.[35] Accordingly, enhanced tumor mutational burden was associated with KALRN mutations in six independent patient cohorts.[44] We hypothesize that the heightened Kalirin levels within the low-TIL No pCR population we observe in our dataset may contribute to treatment resistance through enhanced DNA-damage-repair mechanisms. This could enable improved resilience to chemotherapy treatment and continued immune escape by reduced neoantigen loads. Assessing the post-treatment mutational burden in these tumors would provide useful information to support this hypothesis and an important consideration for future study designs.

Taken together, our results indicate that there is no singular mechanism that can be attributed to treatment resistance, but rather a panel of molecular features. From this panel we identified a treatment-resistant signature that could predict response based on the expression of five proteins (HER2, HER4, ESR, IGF1R, Kalirin). It is important to note that this signature is derived from a limited number of samples and requires further validation at the protein level in additional patient cohorts. Interestingly, we observed that in npCR tumors, the expression levels of this treatment-resistance signature were halfway between the pCR and No pCR tumors (Figures 1A, 1G, and S3B). In addition, we were unable to identify npCR-specific protein signatures. This indicates that the npCR tumors may harbor a mixed cell population, composed of cells that are treatment responsive and resistant. This group of patients likely benefits from the administered therapy but may require additional therapeutic intervention to exploit the therapeutic vulnerabilities of the resistant tumor cells.

We think that this study highlights the feasibility and necessity of clinical proteomics in the study of drug resistance. The

CellPress
OPEN ACCESS

lack of predictive protein biomarkers for treatment outcome despite years of research is troubling and necessitates a different approach.[45] A shift toward system-wide analysis of clinical samples can overcome the challenging translation of *in vitro* findings to patient samples and provide insights into the heavy crosstalk between signaling pathways *in vivo*. In this study, we show that the technological advancements made in recent years allow for great sampling depth of a standard needle biopsy. Because no additional surgery is needed, this approach could be applied to much larger cohorts of patients, paving the path to accurate patient stratification and precision oncology. Further research is needed to gain insights into *in vivo* tumor biology, especially in the field of phosphoproteomics. Our understanding of the biological relevance and clinical significance of phosphosites is lacking, hampering interpretation of phosphoproteomics data. To make advancements in this field, large clinical phosphoproteomics datasets are needed to correlate phosphorylation status with biological outcomes, such as our current study, where we identified a treatment-resistant signature primarily based on our proteome data, and further supported by the phosphoproteomics data.

Limitations of the study

We emphasize that the identified resistance mechanisms require further investigation, especially seeing the small sample size of this study. The aim of this study was to gain insights into resistance mechanisms observed *in vivo*. Some resistance mechanisms were identified in only a very limited number of patients (such as a switch toward OXPHOS). In addition, because all patients within the cohort received both chemotherapy and targeted therapy, we cannot delineate which treatment regimen correlates with our observed resistance signatures, or if our observations are associated with the combined effects from both PTC and TTZ/Ptz treatment together. We acknowledge that these results should be interpreted cautiously and require further study before they can be considered clinically actionable.

STAR★METHODS

Detailed methods are provided in the online version of this paper and include the following:

- KEY RESOURCES TABLE
- RESOURCE AVAILABILITY
  - Lead contact
  - Materials availability
  - Data and code availability
- EXPERIMENTAL MODEL AND SUBJECT DETAILS
- METHOD DETAILS
  - Biopsy preparation
  - Sample lysis and preparation
  - LC-MS/MS analysis
  - Western Blot analysis
- QUANTIFICATION AND STATISTICAL ANALYSIS
  - Preprocessing of datasets
  - Statistics

SUPPLEMENTAL INFORMATION

Supplemental information can be found online at https://doi.org/10.1016/j.xcrm.2023.101203.

ACKNOWLEDGMENTS

We would like to thank Lennart Mulder for technical assistance and the NKI-AVL Core Facility Molecular Pathology & Biobanking (CFMPB) for supplying NKI-AVL Biobank material. D.O.D., K.E.S., and M.A. acknowledge support from the Horizon 2020 program INFRAIA project Epic-XS (Project 823839), and the NWO funded Netherlands Proteomics Center through the National Road Map for Large-scale Infrastructures program X-Omics (Project 184.034.019) of the Netherlands Proteomics Centre.

AUTHOR CONTRIBUTIONS

Conceptualization, D.O.D., K.E.S., and M.A.; methodology, D.O.D., K.E.S., and M.C.L.; investigation, D.O.D., K.E.S., A.P., and M.C.L.; writing – original draft, D.O.D. and K.E.S.; writing – review & editing, D.O.D., K.E.S., G.S.S., J.W., E.H.L., and M.A.; funding acquisition, G.S.S., J.W., and M.A.; resources, G.S.S., J.W., and M.A.; supervision, E.H.L. and M.A.

DECLARATION OF INTERESTS

The authors declare no competing interests.

Received: December 21, 2022
Revised: July 6, 2023
Accepted: August 31, 2023
Published: October 3, 2023

REFERENCES

1. Hu, Z., Fan, C., Oh, D.S., Marron, J.S., He, X., Qaqish, B.F., Livasy, C., Carey, L.A., Reynolds, E., Dressler, L., et al. (2006). The molecular portraits of breast tumors are conserved across microarray platforms. BMC Genom. *7*, 96–12. https://doi.org/10.1186/1471-2164-7-96.
2. Howlader, N., Altekruse, S.F., Li, C.I., Chen, V.W., Clarke, C.A., Ries, L.A.G., and Cronin, K.A. (2014). US incidence of breast cancer subtypes defined by joint hormone receptor and HER2 status. J. Natl. Cancer Inst. *106*, dju055. https://doi.org/10.1093/jnci/dju055.
3. Swain, S.M., Miles, D., Kim, S.B., Im, Y.H., Im, S.A., Semiglazov, V., Ciruelos, E., Schneeweiss, A., Loi, S., Monturus, E., et al. (2020). Pertuzumab, trastuzumab, and docetaxel for HER2-positive metastatic breast cancer (CLEOPATRA): end-of-study results from a double-blind, randomised, placebo-controlled, phase 3 study. Lancet Oncol. *21*, 519–530. https://doi.org/10.1016/S1470-2045(19)30863-0.
4. Swain, S.M., Kim, S.B., Cortés, J., Ro, J., Semiglazov, V., Campone, M., Ciruelos, E., Ferrero, J.M., Schneeweiss, A., Knott, A., et al. (2013). Pertuzumab, trastuzumab, and docetaxel for HER2-positive metastatic breast cancer (CLEOPATRA study): Overall survival results from a randomised, double-blind, placebo-controlled, phase 3 study. Lancet Oncol. *14*, 461–471. https://doi.org/10.1016/S1470-2045(13)70130-X.
5. Swain, S.M., Baselga, J., Kim, S.-B., Ro, J., Semiglazov, V., Campone, M., Ciruelos, E., Ferrero, J.-M., Schneeweiss, A., Heeson, S., et al. (2015). Pertuzumab, Trastuzumab, and Docetaxel in HER2-Positive Metastatic Breast Cancer. N. Engl. J. Med. *372*, 724–734. https://doi.org/10.1056/nejmoa1413513.
6. Van Der Voort, A., Van Ramshorst, M.S., Van Werkhoven, E.D., Mandjes, I.A., Kemper, I., Vulink, A.J., Oving, I.M., Honkoop, A.H., Tick, L.W., Van De Wouw, A.J., et al. (2021). Three-Year Follow-up of Neoadjuvant Chemotherapy with or without Anthracyclines in the Presence of Dual ERBB2 Blockade in Patients with ERBB2-Positive Breast Cancer: A Secondary Analysis of the TRAIN-2 Randomized, Phase 3 Trial. JAMA Oncol. *7*, 978–984, –7. https://doi.org/10.1001/jamaoncol.2021.1371.

7. Pernas, S., and Tolaney, S.M. (2019). HER2-positive breast cancer: new therapeutic frontiers and overcoming resistance. Ther. Adv. Med. Oncol. *11*, 1758835919833519–16. https://doi.org/10.1177/1758835919833519.

8. Tanioka, M., Fan, C., Parker, J.S., Hoadley, K.A., Hu, Z., Li, Y., Hyslop, T.M., Pitcher, B.N., Soloway, M.G., Spears, P.A., et al. (2018). Integrated analysis of RNA and DNA from the phase III trial CALGB 40601 identifies predictors of response to trastuzumab-based neoadjuvant chemotherapy in HER2-positive breast cancer. Clin. Cancer Res. *24*, 5292–5304. https://doi.org/10.1158/1078-0432.CCR-17-3431.

9. Krug, K., Jaehnig, E.J., Satpathy, S., Blumenberg, L., Karpova, A., Anurag, M., Miles, G., Mertins, P., Geffen, Y., Tang, L.C., et al. (2020). Proteogenomic Landscape of Breast Cancer Tumorigenesis and Targeted Therapy. Cell *183*, 1436–1456.e31. https://doi.org/10.1016/j.cell.2020.10.036.

10. Carey, L.A., Berry, D.A., Cirrincione, C.T., Barry, W.T., Pitcher, B.N., Harris, L.N., Ollila, D.W., Krop, I.E., Henry, N.L., Weckstein, D.J., et al. (2016). Molecular heterogeneity and response to neoadjuvant human epidermal growth factor receptor 2 targeting in CALGB 40601, a randomized phase III trial of paclitaxel plus trastuzumab with or without lapatinib. J. Clin. Oncol. *34*, 542–549. https://doi.org/10.1200/JCO.2015.62.1268.

11. van Ramshorst, M.S., van der Voort, A., van Werkhoven, E.D., Mandjes, I.A., Kemper, I., Dezentjé, V.O., Oving, I.M., Honkoop, A.H., Tick, L.W., van de Wouw, A.J., et al. (2018). Neoadjuvant chemotherapy with or without anthracyclines in the presence of dual HER2 blockade for HER2-positive breast cancer (TRAIN-2): a multicentre, open-label, randomised, phase 3 trial. Lancet Oncol. *19*, 1630–1640. https://doi.org/10.1016/S1470-2045(18)30570-9.

12. Vu, T., and Claret, F.X. (2012). Trastuzumab: Updated mechanisms of action and resistance in breast cancer. Front. Oncol. *2*, 62. https://doi.org/10.3389/fonc.2012.00062.

13. Vernieri, C., Milano, M., Brambilla, M., Mennitto, A., Maggi, C., Cona, M.S., Prisciandaro, M., Fabbroni, C., Celio, L., Mariani, G., et al. (2019). Resistance mechanisms to anti-HER2 therapies in HER2-positive breast cancer: Current knowledge, new research directions and therapeutic perspectives. Crit. Rev. Oncol. Hematol. *139*, 53–66. https://doi.org/10.1016/j.critrevonc.2019.05.001.

14. Satpathy, S., Jaehnig, E.J., Krug, K., Kim, B.J., Saltzman, A.B., Chan, D.W., Holloway, K.R., Anurag, M., Huang, C., Singh, P., et al. (2020). Microscaled proteogenomic methods for precision oncology. Nat. Commun. *11*, 532. https://doi.org/10.1038/s41467-020-14381-2.

15. Wolf, D.M., Yau, C., Wulfkuhle, J., Brown-Swigart, L., Gallagher, R.I., Lee, P.R.E., Zhu, Z., Magbanua, M.J., Sayaman, R., O'Grady, N., et al. (2022). Redefining breast cancer subtypes to guide treatment prioritization and maximize response: Predictive biomarkers across 10 cancer therapies. Cancer Cell *40*, 609–623.e6. https://doi.org/10.1016/j.ccell.2022.05.005.

16. Ekyalongo, R.C., and Yee, D. (2017). Revisiting the IGF-1R as a breast cancer target. npj Precis. Oncol. *1*, 14–6. https://doi.org/10.1038/s41698-017-0017-y.

17. Adams, T.E., Epa, V.C., Garrett, T.P., and Ward, C.W. (2000). Structure and function of the type 1 insulin-like growth factor receptor. Cell. Mol. Life Sci. *57*, 1050–1093. https://doi.org/10.1007/PL00000744.

18. Fagan, D.H., and Yee, D. (2008). Crosstalk between IGF1R and estrogen receptor signaling in breast cancer. J. Mammary Gland Biol. Neoplasia *13*, 423–429.

19. McDermott, M.S.J., Canonici, A., Ivers, L., Browne, B.C., Madden, S.F., O'Brien, N.A., Crown, J., and O'Donovan, N. (2017). Dual inhibition of IGF1R and ER enhances response to trastuzumab in HER2 positive breast cancer cells. Int. J. Oncol. *50*, 2221–2228. https://doi.org/10.3892/ijo.2017.3976.

20. Hanahan, D., and Weinberg, R.A. (2011). Hallmarks of cancer: The next generation. Cell *144*, 646–674. https://doi.org/10.1016/j.cell.2011.02.013.

21. Faubert, B., Solmonson, A., and DeBerardinis, R.J. (2020). Metabolic reprogramming and cancer progression. Science *368*, eaaw5473. https://doi.org/10.1126/science.aaw5473.

22. Guerra, F., Arbini, A.A., and Moro, L. (2017). Mitochondria and cancer chemoresistance. Biochim. Biophys. Acta Bioenerg. *1858*, 686–699. https://doi.org/10.1016/j.bbabio.2017.01.012.

23. Levin, E.R., and Hammes, S.R. (2016). Nuclear receptors outside the nucleus : receptors. Nat. Rev. Mol. Cell Biol. *17*, 783–797. https://doi.org/10.1038/nrm.2016.122.Nuclear.

24. Thirupathi, A., and Chang, Y.Z. (2019). Role of AMPK and its molecular intermediates in subjugating cancer survival mechanism. Life Sci. *227*, 30–38. https://doi.org/10.1016/j.lfs.2019.04.039.

25. Chaube, B., Malvi, P., Singh, S.V., Mohammad, N., Viollet, B., and Bhat, M.K. (2015). AMPK maintains energy homeostasis and survival in cancer cells via regulating p38/PGC-1α-mediated mitochondrial biogenesis. Cell Death Dis. *1*, 15063–11. https://doi.org/10.1038/cddiscovery.2015.63.

26. Ito, D., Walker, J.R., Thompson, C.S., Moroz, I., Lin, W., Veselits, M.L., Hakim, A.M., Fienberg, A.A., and Thinakaran, G. (2004). Characterization of Stanniocalcin 2, a Novel Target of the Mammalian Unfolded Protein Response with Cytoprotective Properties. Mol. Cell Biol. *24*, 9456–9469. https://doi.org/10.1128/mcb.24.21.9456-9469.2004.

27. Fazio, E.N., DiMattia, G.E., Chadi, S.A., Kernohan, K.D., and Pin, C.L. (2011). Stanniocalcin 2 alters PERK signalling and reduces cellular injury during cerulein induced pancreatitis in mice. BMC Cell Biol. *12*, 17. https://doi.org/10.1186/1471-2121-12-17.

28. Hsu, S.K., Chiu, C.C., Dahms, H.U., Chou, C.K., Cheng, C.M., Chang, W.T., Cheng, K.C., Wang, H.M.D., and Lin, I.L. (2019). Unfolded protein response (UPR) in survival, dormancy, immunosuppression, metastasis, and treatments of cancer cells. Int. J. Mol. Sci. *20*, 2518. https://doi.org/10.3390/ijms20102518.

29. Wu, S., Ma, S., Yin, X., Yi, P., and Liu, J. (2019). An integrated PKD1-dependent signaling network amplifies IRE1 prosurvival signaling. J. Biol. Chem. *294*, 11119–11130. https://doi.org/10.1074/jbc.RA118.003311.

30. Avril, T., Vauléon, E., and Chevet, E. (2017). Endoplasmic reticulum stress signaling and chemotherapy resistance in solid cancers. Oncogenesis *6*, e373. https://doi.org/10.1038/oncsis.2017.72.

31. Reich, S., Nguyen, C.D.L., Has, C., Steltgens, S., Soni, H., Coman, C., Freyberg, M., Bichler, A., Seifert, N., Conrad, D., et al. (2020). A multi-omics analysis reveals the unfolded protein response regulon and stress-induced resistance to folate-based antimetabolites. Nat. Commun. *11*, 2936–15. https://doi.org/10.1038/s41467-020-16747-y.

32. Bahar, E., Kim, J.Y., and Yoon, H. (2019). Chemotherapy resistance explained through endoplasmic reticulum stress-dependent signaling. Cancers *11*, 338–20. https://doi.org/10.3390/cancers11030338.

33. Danaher, P., Warren, S., Dennis, L., D'Amico, L., White, A., Disis, M.L., Geller, M.A., Odunsi, K., Beechem, J., and Fling, S.P. (2017). Gene expression markers of Tumor Infiltrating Leukocytes. J. Immunother. Cancer *5*, 18. https://doi.org/10.1186/s40425-017-0215-8.

34. Remmers, C., Sweet, R.A., and Penzes, P. (2014). Abnormal kalirin signaling in neuropsychiatric disorders. Brain Res. Bull. *103*, 29–38. https://doi.org/10.1016/j.brainresbull.2013.12.006.

35. Li, M., Ma, Y., Zhong, Y., Liu, Q., Chen, C., Qiang, L., and Wang, X. (2020). KALRN mutations promote antitumor immunity and immunotherapy response in cancer. J. Immunother. Cancer *8*, e000293. https://doi.org/10.1136/jitc-2019-000293.

36. Cardoso, F., Kyriakides, S., Ohno, S., Penault-Llorca, F., Poortmans, P., Rubio, I.T., Zackrisson, S., and Senkus, E.; ESMO Guidelines Committee. Electronic address: clinicalguidelines@esmo.org (2019). Early breast cancer: ESMO Clinical Practice Guidelines for diagnosis, treatment and follow-up. Ann. Oncol. *30*, 1194–1220. https://doi.org/10.1093/annonc/mdz173.

37. Clark, A.S., Yau, C., Wolf, D.M., Petricoin, E.F., van 't Veer, L.J., Yee, D., Moulder, S.L., Wallace, A.M., Chien, A.J., Isaacs, C., et al. (2021). Neoadjuvant T-DM1/pertuzumab and paclitaxel/trastuzumab/pertuzumab for

CellPress
OPEN ACCESS

HER2+ breast cancer in the adaptively randomized I-SPY2 trial. Nat. Commun. *12*, 6428. https://doi.org/10.1038/s41467-021-26019-y.

38. Hammond, M.E.H., Hayes, D.F., Dowsett, M., Allred, D.C., Hagerty, K.L., Badve, S., Fitzgibbons, P.L., Francis, G., Goldstein, N.S., Hayes, M., et al. (2010). American society of clinical oncology/college of american pathologists guideline recommendations for immunohistochemical testing of estrogen and progesterone receptors in breast cancer. J. Clin. Oncol. *28*, 2784–2795. https://doi.org/10.1200/JCO.2009.25.6529.
39. Brockhoff, G. (2019). Target HER four in breast cancer? Oncotarget *10*, 3147–3150. https://doi.org/10.18632/oncotarget.26867.
40. Canfield, K., Li, J., Wilkins, O.M., Morrison, M.M., Ung, M., Wells, W., Williams, C.R., Liby, K.T., Vullhorst, D., Buonanno, A., et al. (2015). Receptor tyrosine kinase ERBB4 mediates acquired resistance to ERBB2 inhibitors in breast cancer cells. Cell Cycle *14*, 648–655. https://doi.org/10.4161/15384101.2014.994966.
41. Mohd Nafi, S.N., Generali, D., Kramer-Marek, G., Gijsen, M., Strina, C., Cappelletti, M., Andreis, D., Haider, S., Li, J.-L., Bridges, E., et al. (2014). Nuclear HER4 mediates acquired resistance to trastuzumab and is associated with poor outcome in HER2 positive breast cancer. Oncotarget *5*, 5934–5949. https://doi.org/10.18632/oncotarget.1904.
42. Reinholz, M.M., Chen, B., Dueck, A.C., Tenner, K., Ballman, K., Riehle, D., Jenkins, R.B., Geiger, X.J., McCullough, A.E., and Perez, E.A. (2017). IGF1R protein expression is not associated with differential benefit to concurrent trastuzumab in early-stage HER2+ breast cancer from the North Central Cancer Treatment Group (Alliance) adjuvant trastuzumab trial N9831. Clin. Cancer Res. *23*, 4203–4211. https://doi.org/10.1158/1078-0432.CCR-15-0574.
43. Schmid, P., Salgado, R., Park, Y.H., Muñoz-Couselo, E., Kim, S.B., Sohn, J., Im, S.A., Foukakis, T., Kuemmel, S., Dent, R., et al. (2020). Pembrolizumab plus chemotherapy as neoadjuvant treatment of high-risk, early-stage triple-negative breast cancer: results from the phase 1b open-label, multicohort KEYNOTE-173 study. Ann. Oncol. *31*, 569–581. https://doi.org/10.1016/j.annonc.2020.01.072.
44. Miao, Y.R., Liu, C.J., Hu, H., Yang, M., and Guo, A.Y. (2022). Biological Pathway-Derived TMB Robustly Predicts the Outcome of Immune Checkpoint Blockade Therapy. Cells *11*. https://doi.org/10.3390/cells11182802.
45. Gingras, I., Gebhart, G., De Azambuja, E., and Piccart-Gebhart, M. (2017). HER2-positive breast cancer is lost in translation: Time for patient-centered research. Nat. Rev. Clin. Oncol. *14*, 669–681. https://doi.org/10.1038/nrclinonc.2017.96.
46. van Ramshorst, M.S., van Werkhoven, E., Honkoop, A.H., Dezentjé, V.O., Oving, I.M., Mandjes, I.A., Kemper, I., Smorenburg, C.H., Stouthard, J.M., Linn, S.C., et al. (2016). Toxicity of dual HER2-blockade with pertuzumab added to anthracycline versus non-anthracycline containing chemotherapy as neoadjuvant treatment in HER2-positive breast cancer: The TRAIN-2 study. Breast *29*, 153–159. https://doi.org/10.1016/j.breast.2016.07.017.
47. Post, H., Penning, R., Fitzpatrick, M.A., Garrigues, L.B., Wu, W., Macgillavry, H.D., Hoogenraad, C.C., Heck, A.J.R., and Altelaar, A.F.M. (2017). Robust, Sensitive, and Automated Phosphopeptide Enrichment Optimized for Low Sample Amounts Applied to Primary Hippocampal Neurons. J. Proteome Res. *16*, 728–737. https://doi.org/10.1021/acs.jproteome.6b00753.
48. Krug, K., Mertins, P., Zhang, B., Hornbeck, P., Raju, R., Ahmad, R., Szucs, M., Mundt, F., Forestier, D., Jane-Valbuena, J., et al. (2019). A Curated Resource for Phosphosite-specific Signature Analysis. Mol. Cell. Proteomics *18*, 576–593. https://doi.org/10.1074/mcp.TIR118.000943.
49. Subramanian, A., Tamayo, P., Mootha, V.K., Mukherjee, S., Ebert, B.L., Gillette, M.A., Paulovich, A., Pomeroy, S.L., Golub, T.R., Lander, E.S., et al. (2005). Gene Set Enrichment Analysis: A Knowledge-Based Approach for Interpreting Genome-wide Expression Profiles.
50. Mootha, V.K., Lindgren, C.M., Eriksson, K.-F., Subramanian, A., Sihag, S., Lehar, J., Puigserver, P., Carlsson, E., Ridderstråle, M., Laurila, E., et al. (2003). A R T I C L E S PGC-1α-responsive genes involved in oxidative phosphorylation are coordinately downregulated in human diabetes.
51. Zhou, Y., Zhou, B., Pache, L., Chang, M., Khodabakhshi, A.H., Tanaseichuk, O., Benner, C., and Chanda, S.K. (2019). Metascape provides a biologist-oriented resource for the analysis of systems-level datasets. Nat. Commun. *10*, 1523. https://doi.org/10.1038/s41467-019-09234-6.

STAR★METHODS

KEY RESOURCES TABLE

REAGENT	SOURCE	IDENTIFIER
Biological samples		
Human IBC tumor samples	TRAIN2 clinical trial	https://clinicaltrials.gov/ct2/show/NCT01996267
Chemicals, peptides and recombinant proteins		
Sodium deoxycholate (SDC)	Sigma Aldrich	MFCD00064139
Tris(2-carboxyethyl) phosphine hydrochloride (TCEP)	Sigma Aldrich	MFCD00145469
Chloroacetamide (CAA)	Sigma Aldrich	MFCD0008027
TRIS	Sigma Aldrich	
PhosSTOP	Merck	4906837001
cOmplete, Mini, EDTA-free Protease Inhibitor Cocktail	Merck	11836170001
TMT 11-plex reagent	Thermo Scientific	Cat# A34808
Trypsin	Thermo Scientific	Prod# 90057S
LysC	Wako	125–05061
Criterion XT Gels	Bio-Rad	3450130
PVDF Membranes	Bio-Rad	1620177
XT MOPS	Bio-Rad	1610788
Pierce™ ECL Plus Western Blotting Substrate	Thermo Scientific	Cat#32132
Antibodies		
Estrogen Receptor α (D8H8) Rabbit mAb	Cell Signaling	#8644
HER2/ErbB2 (D8F12) XP® Rabbit mAb	Cell Signaling	#4290T
Anti-Rabbit IgG, HRP-linked Ab	Cell Signaling	#7074
β-actin (14E5) Rabbit mAb	Cell Signaling	#4970S
Critical commercial assays		
AssayMap Cartridge Rack, Fe(III)-NTA 5 mL	Agilent Technologies	Cat#G5496-60085
Kinetex 5u Evo C18 100A 150x2.1mm	Phenomenex	00F-4633-AN
Poroshell 120 EC-C18 2.7 micron	Agilent Technologies	AG699975-902
AssayMAP Cartridge Rack C18 5ul	Agilent Technologies	Cat# 5190-6532
Sep-Pak C18 1 cc Vac Cartridge	Waters	WAT023590
Bradford Protein Assay	Bio-Rad	5000006
Deposited data		
Raw data	PRIDE	PXD034643
Proteomics search results	PRIDE	PXD034643
Software and algorithms		
Proteome Discoverer 2.2	Thermo Scientific	OPTON-30812
GraphPad Prism 9.3.0	Graphpad Software Inc	https://www.graphpad.com/scientific-software/prism/
R v4.3.3	https://www.r-project.org/	https://www.r-project.org/
RStudio (v2022.02.1 461)	RStudio, PBC	https://www.rstudio.com/

RESOURCE AVAILABILITY

Lead contact

Further information and requests for resources and reagents should be directed to and will be fulfilled by the lead contact, Maarten Altelaar (m.altelaar@uu.nl)

Materials availability

The study did not generate new unique reagents.

CellPress
OPEN ACCESS

Data and code availability

- The mass spectrometry proteomics and phosphoproteomics data used in this study have been deposited to ProteomeXchange Consortium via the PRIDE repository under the identification code PXD034643.
- This study did not generate original code.
- Any additional information required to reanalyses the data reported in this paper is available from the lead contact upon request.

EXPERIMENTAL MODEL AND SUBJECT DETAILS

Forty-five patient biopsies of treatment-naive primary breast tumors were obtained from patients enrolled in the TRAIN-2 clinical trial (NCT01996267)[46] and were collected at the Netherlands Cancer Institute between 2013 and 2016. The use of biobank samples for this study was approved by the institutional review board (IRB) of the Netherlands Cancer Institute under number CFMPB672. All patients received neo-adjuvant treatment consisting of paclitaxel, carboplatin, TTZ, Ptz (PTC-Ptz). The proteomics study as presented here was performed on remaining frozen biopsies in the institute's biobank. After careful consideration, we were able to include 45 high quality biopsies for proteomics studies. As the current study is primarily explorative in nature we did not perform a power calculation.

METHOD DETAILS

Biopsy preparation

Patient pre-treatment biopsies were taken with a 14G needle and flash frozen in liquid nitrogen. Biopsies were sliced and approximately one-third of the material was allocated for this study (ca. 6–12 mg of tissue). Only biopsies with >60% tumor cells were selected for analysis. Patient information and tumor details can be found in SI Table S1.

Sample lysis and preparation

Tissue lysis was performed in a 1% (w/v) sodium deoxycholate lysis buffer containing 10mM TCEP, 100mM TRIS, 40mM chloroacetamide, and protease inhibitor and phosphatase inhibitor tablet. Tissue slices were homogenized by multiple freeze-thaw cycles in combination with grinding by pestle in a 1.7mL sample tube in lysis buffer. Samples were then boiled for 5 min at 95°C and sonicated in a Bioruptor 300 (Diagenode) water bath for 30 min using 30 s cycles. Protein quantification was performed using Bradford Protein Assay (Bio-Rad) and 150ug of protein per sample was digested overnight with Lys-C (1:75) and trypsin (1:25) at 37°C. Samples were acidified and desalted using C18 cartridges on the AssayMap BRAVO Platform (Agilent Technologies). The TMT reference channel was made by pooling 25 μg of peptide from each sample. Samples were dried and resuspended in 50mM HEPES buffer, randomized, and then labeled with 10-plex TMT reagent (Thermo Scientific) in a 1:2 ratio (peptide: label) for 1.5 h at room temperature. TMT labeling reaction was quenched using a 5% hydroxylamine solution before samples were mixed in equal ratios to generate 5 complete TMT sets. Pooled samples were then desalted using Sep-Pac C18 cartridges (Waters), and fractionated on a high-pH reversed-phase C18 column (Kinetex 5u Evo C18 100A, 150 × 2.1mm, Phenomenex) coupled to an Agilent 1100 series HPLC over a 60 min gradient. Fractions were concatenated to 20 fractions for proteome analysis and further pooled to 10 fractions for phosphoproteome enrichment. Phosphoproteome samples were enrichened using Fe(III)-IMAC cartridges on the AssayMap BRAVO platform (Agilent Technologies) following the method described previously.[47]

LC-MS/MS analysis

Fractionated TMT samples were analyzed by nanoLC-MS/MS on a Q Exactive HF-X mass spectrometer (Thermo Scientific) in-line with an Agilent 1290 HPLC system possessing a Reprosil pur C18 trap column (100 μm × 2 cm, 3 μm, Dr. Maisch) and a Poroshell 120 EC C18 analytical column (75 μm × 50 cm, 2.7 μm, Agilent Technologies). Samples were trapped for 5 min at a flow rate of 0.05 mL/min in 100% buffer A (0.1% FA) followed by elution with buffer B (0.1% FA, 80% ACN) at a flowrate of 300 nL/min over an LC gradient of 65 min (15%–45% B) for proteome fractions and a 95 min gradient (9%–35% B) for phosphoproteome fractions. MS settings were as follows: full MS scans (375–1500 m/z) were acquired at 60,000 resolution with an AGC target of 3e6 charges and max injection time of 20 msec. HCD MS2 spectra were generated for the top 12 precursors using 45,000 resolution, 1e5 AGC target, a max injection time of 80 msec, a fixed first mass of 120m/z, and a normalised collision energy of 32%. MS2 isolation windows were 0.7 Th for proteome samples and 1.2 Th for phosphoproteome samples.

Western Blot analysis

Tissue lysates containing a total of 40ug proteins were loaded on Criterion XT Gels (Bio-Rad). SDS-PAGE Electrophoresis was performed in XT-MOPS running buffer (Bio-Rad), and afterward, proteins were transferred into Immun-Blot PVDF Membranes (Bio-Rad). After blocking with 5% skim milk at room temperature for 1 h, membranes were incubated with primary antibodies at 4°C overnight. The primary antibodies against Estrogen receptor α, Her-2 and β-Actin (Cell Signaling Technology) were diluted 1:1000 with tris-buffered saline containing 1% Tween 20 (TBST). Incubation with secondary horseradish peroxidase-conjugated anti-rabbit antibody

(Cell Signaling Technology) in 1:3000 dilution was performed at RT for 1h. The chemiluminescence detection was performed with Pierce ECL Plus Western Blotting Substrate (Thermo Fisher Scientific) and detected with Amersham Imager 600 (GE Healthcare, UK).

QUANTIFICATION AND STATISTICAL ANALYSIS

Preprocessing of datasets

Raw data files were processed with Proteome Discover 2.2 (Thermo Scientific) using a Sequest HT search against the Swissprot human database. Results were filtered using a 1% FDR cut off at the protein and peptide level. TMT fragment ions were quantified using summed abundances with PSM filters requiring an S/N $\geq$ 10 and an isolation interference cut off of 35% or 50% (proteome and phosphoproteome respectively). Normalised protein and peptide abundances were extracted from PD2.2 and further scaled and analyzed using Rstudio. Data was normalised by the pooled reference, Log2 transformed and normalised by median subtraction. Phosphoproteome data was filtered to include only phosphopeptides with a class I phosphosite localization (ptmRS score >0.75). Phosphopeptides containing identical phosphorylation site localizations with different methionine oxidation sates or peptide missed cleavages were summed together to generate one quantitative value per unique phosphosite. Phosphosites quantified in peptides with different phosphorylation multiplicity states (i.e., doubly or singly phosphorylated) were not combined together and left as separate quantified values.

Statistics

To compare three means, a one-way ANOVA test was used (using the aov-function, followed by TukeyHSD-function in Rstudio). To compare two means, a two-sample T-test was used (using the t.test-function in Rstudio). A p value below 0.05 was regarded as statistically significant. Correlation analysis was performed by Pearson correlation using cor-function in RStudio. PTM-SEA analysis was performed using PTMsigDB.[48] Pathway enrichment analysis was performed using GSEA[49,50] or Metascape using a custom background of all detected proteins in our MS analysis.[51] Heatmaps were generated using pheatmap-function in Rstudio. Data was z-scored and Euclidean distance was used for clustering. ROC analysis was performed using pROC-function in RStudio, comparing No pCR and pCR patients only (near pCR patients were removed from the dataset prior to analysis). ROC curves for individual proteins were generated using roc-function, ROC curve of the combination of proteins was generated using multiclass.roc-function.

Cell Reports Medicine, Volume *4*

Supplemental information

Deep (phospho)proteomics profiling of pre-treatment needle biopsies identifies signatures of treatment resistance in HER2$^+$ breast cancer

Donna O. Debets, Kelly E. Stecker, Anastasia Piskopou, Marte C. Liefaard, Jelle Wesseling, Gabe S. Sonke, Esther H. Lips, and Maarten Altelaar

Supplementary Table 1. Patient information. Related to STAR Methods.

Patient	*Histology*	*Tumour grade*	*ER (%)*	*PR (%)*	*HER2 score*	*Treatment outcome*	*Tumour (%)*	*TMT pool_label*
1	ID	2	50	0	3+	pCR	80	Pool1_126
2	ID	2	80	80	3+	pCR	80	Pool1_127N
3	ID	3	0	0	3+	pCR	60	Pool1_127C
4	ID	2	80	5	3+	pCR	70	Pool1_128N
5	ID	2	25	0	3+	pCR	70	Pool1_128C
6	ID	3	0	0	3+	pCR	70	Pool1_129N
7	ID	3	100	1	2+	No pCR	80	Pool1_129C
8	ID	2	70	70	3+	pCR	60	Pool1_130N
9	ID	3	60	70	3+	pCR	80	Pool1_130C
10	ID	3	100	5	3+	No pCR	80	Pool1_131N
11	ID	2	100	70	3+	pCR	80	Pool2_126
12	ID	3	20	0	3+	pCR	80	Pool2_127N
13	IL	3	100	40	3+	npCR	80	Pool2_127C
14	ID	2	40	0	3+	pCR	60	Pool2_128N
15	ID	2	0	0	3+	pCR	70	Pool2_128C
16	ID	2	0	0	3+	pCR	60	Pool2_129N
17	ID	2	100	0	3+	npCR	70	Pool2_129C
18	ID	3	60	5	2+	npCR	60	Pool2_130N
19	ID	2	100	100	3+	pCR	80	Pool2_130C
20	IL	2	100	0	2+	No pCR	80	Pool2_131N
21	ID	2	100	0	3+	pCR	80	Pool3_126
22	ID	3	100	0	2+	pCR	80	Pool3_127N
23	ID	2	100	1	2+	pCR	70	Pool3_127C
24	ID	3	80	70	3+	npCR	70	Pool3_128N
25	ID	2	0	0	3+	pCR	60	Pool3_128C
26	ID	3	0	0	3+	pCR	60	Pool3_129N
27	IL	3	90	0	3+	pCR	80	Pool3_129C
28	ID	3	90	100	3+	npCR	70	Pool3_130N
29	ID	3	100	60	2+	No pCR	80	Pool3_130C
30	ID	2	90	100	3+	pCR	90	Pool4_126
31	ID	3	90	40	3+	pCR	60	Pool4_127C
32	ID	3	10	0	2+	pCR	80	Pool4_128N
33	ID	2	100	100	3+	pCR	70	Pool4_128C
34	MA	2	100	30	3+	pCR	80	Pool4_129N
35	ID	2	0	0	3+	pCR	60	Pool4_129C
36	ID	3	0	0	3+	npCR	60	Pool4_130N
37	ID	3	100	20	3+	No pCR	80	Pool5_126
38	ID	2	50	100	3+	No pCR	70	Pool5_127N
39	ID	3	100	5	3+	No pCR	80	Pool5_127C
40	ID	2	100	90	3+	npCR	60	Pool5_128N
41	ID	3	100	90	3+	No pCR	70	Pool5_128C
42	MA	2/3	90	20	3+	No pCR	60	Pool5_129N
43	ID	3	100	0	3+	No pCR	80	Pool5_129C
44	ID	2	100	40	3+	No pCR	60	Pool5_130N
45	ID	3	100	1	3+	npCR	60	Pool5_131N

ID: invasive ductal
IL: invasive lobular
MA: Mucinous adenocarcinoma

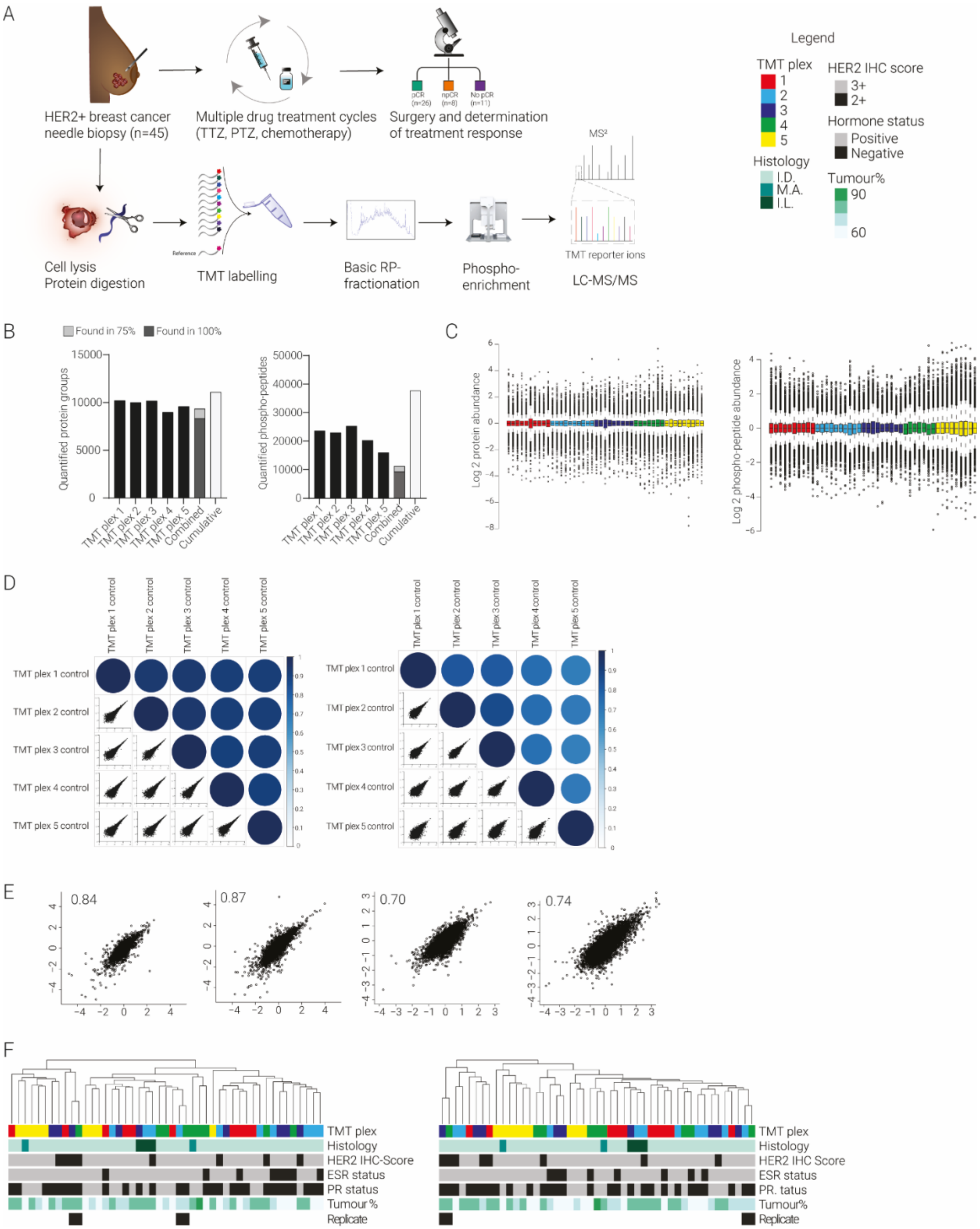

Supplementary Figure 1. Experimental workflow and data quality. Related to STAR Methods. A) 45 HER2+ breast cancer biopsies were collected by needle biopsy procedure. Afterwards, patients received a combination of targeted therapy (Trastuzumab and Pertuzumab) and chemotherapy, followed by surgery. Treatment response was determined based on the absence or presence of remaining tumour cells in the breast and lymph node. Patients were

classified as pCR (pathological complete response), npCR (near pathological complete response) or No pCR (no pathological complete response). The treatment-naive biopsies were used for the (phospho)proteomics analysis. Tumour cells were lysed, proteins extracted and digested. The derived peptides were labelled by Tandem Mass Tag (TMT). Peptides were fractionated by basic reversed-phase chromatography and phosphorylated peptides were enriched prior to LC-MS/MS analysis. B) Number of protein groups (left) and phopsho-peptides (right) quantified. C) Boxplot of median normalised samples of the protein data (left) and phospho-data (right). D) Correlation plot of the inter-plex TMT reference sample of the Log 2 protein abundance data (left) and Log 2 phosphopeptide abundance data (right). E) Correlation of replicate patient samples (Pearson correlation) of protein (left two plots) and phospho-data (right two plots). Scales represent Log2 abundances normalised to the pooled reference channel. F) Unsupervised clustering of all proteins (left) or all phosphopeptides (right) showed grouping of replicate samples. Grouping was not driven by tumour% or TMT-plex.

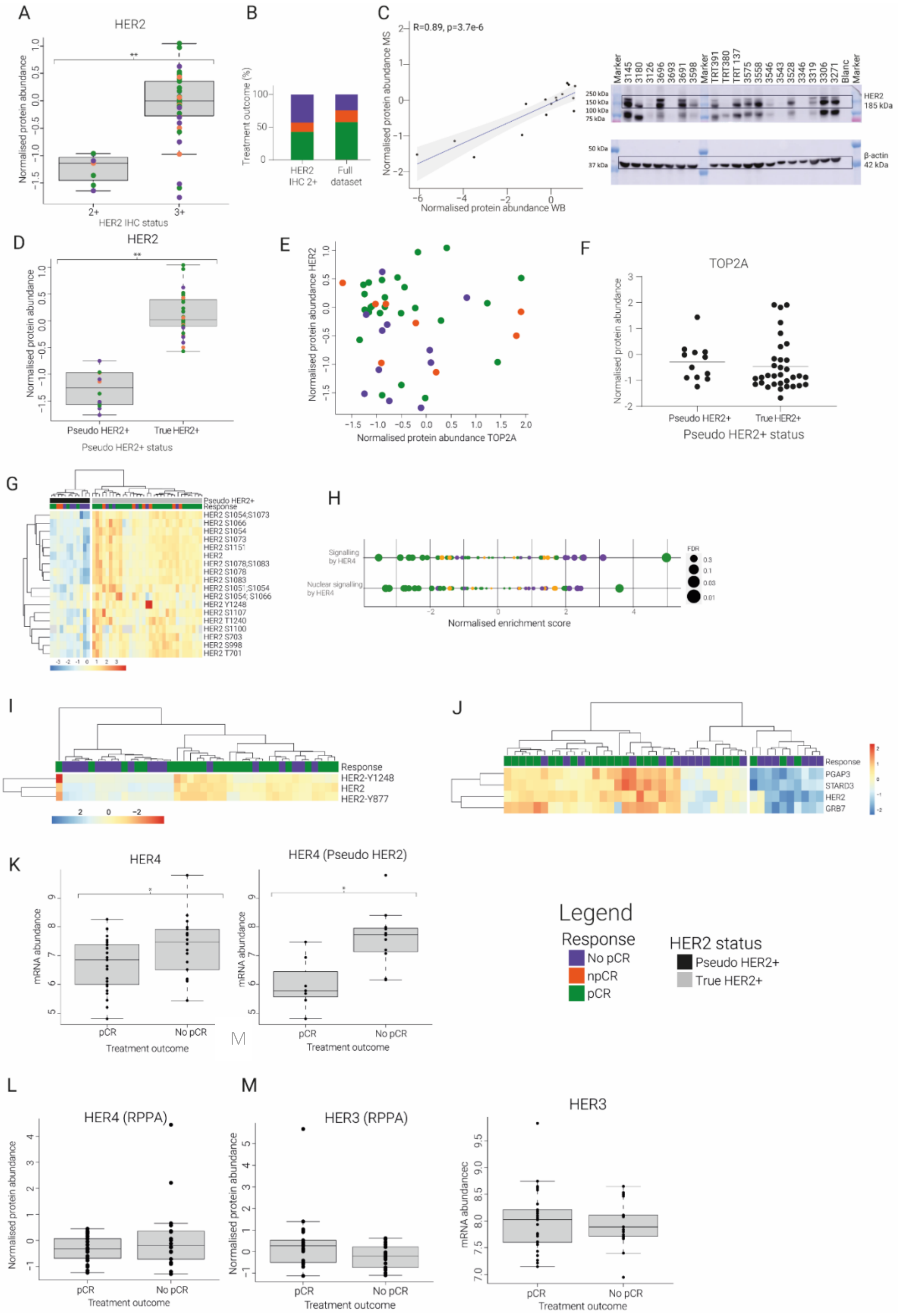
A
HER2
Normalised protein abundance
2+
3+
HER2 IHC status
B
Treatment outcome (%)
HER2 IHC 2+
Full dataset
C
R=0.89, p=3.7e-6
Normalised protein abundance MS
Normalised protein abundance WB
Marker
3145
3180
3126
3696
3693
3691
3598
Marker
TRT391
TRT380
TRT 137
3575
3558
3546
3543
3528
3346
3319
3306
3271
Blanc
Marker
250 kDa
150 kDa
100 kDa
75 kDa
HER2
185 kDa
50 kDa
37 kDa
β-actin
42 kDa
D
HER2
Normalised protein abundance
Pseudo HER2+
True HER2+
Pseudo HER2+ status
E
Normalised protein abundance HER2
Normalised protein abundance TOP2A
F
TOP2A
Normalised protein abundance
Pseudo HER2+
True HER2+
Pseudo HER2+ status
G
Pseudo HER2+
Response
HER2 S1054;S1073
HER2 S1066
HER2 S1054
HER2 S1073
HER2 S1151
HER2
HER2 S1078;S1083
HER2 S1078
HER2 S1083
HER2 S1051;S1054
HER2 S1054; S1066
HER2 Y1248
HER2 S1107
HER2 T1240
HER2 S1100
HER2 S703
HER2 S998
HER2 T701
H
Signalling by HER4
Nuclear signalling by HER4
FDR
0.3
0.1
0.03
0.01
Normalised enrichment score
I
Response
HER2-Y1248
HER2
HER2-Y877
J
Response
PGAP3
STARD3
HER2
GRB7
K
HER4
mRNA abundance
pCR
No pCR
Treatment outcome
HER4 (Pseudo HER2)
mRNA abundance
pCR
No pCR
Treatment outcome
Legend
Response
No pCR
npCR
pCR
HER2 status
Pseudo HER2+
True HER2+
L
HER4 (RPPA)
Normalised protein abundance
pCR
No pCR
Treatment outcome
M
HER3 (RPPA)
Normalised protein abundance
pCR
No pCR
Treatment outcome
HER3
mRNA abundancec
pCR
No pCR
Treatment outcome

Supplementary Figure 2. Pseudo HER2 signature is associated with poor treatment response. Related to Figure 1. A) Boxplot of HER2 protein expression shows significantly increased abundance amongst the HER2 IHC 3+ tumours compared to the 2+ tumours. The lowest HER2 expression levels however were found amongst IHC 3+ tumours. B) pCR rate (%) amongst the HER2 IHC2+ tumours (n=7) compared to the full dataset (n=45). C) Correlation plot of HER2 protein expression levels as established by WB compared to MS and the WB raw data. WB HER2 band intensities are normalized to actin loading control. Box indicates quantified bands. D) Boxplot of HER2 protein expression by HER2-status shows significant downregulation of HER2 within the Pseudo HER2+ subgroup; tumours within the Pseudo HER2+ subgroup have the lowest HER2 expression levels. E) Correlation plot between HER2 protein abundance and TOP2A protein abundance coloured by treatment outcome. F) TOP2A protein abundance between Pseudo HER2+ subgroup and true HER2+ subgroup. G) Heatmap of unsupervised clustering of all phosphosites on HER2 shows clustering of all Pseudo HER2+ tumours together. H) Normalised Enrichment Score of PTM-SEA analysis for HER4 signalling. Dot size represents FDR and colour represent patient outcome. I) Heatmap of unsupervised clustering of RPPA HER2 protein and phosphosites shows enrichment of treatment resistant tumours in a cluster with low HER2 and HER2-phosphosite levels. J) Heatmap of unsupervised clustering of PGAP3, STARD3, HER2 and GRB7 mRNA data shows enrichment of treatment resistant tumours with low abundance of all proteins. K) Boxplot of HER4 mRNA levels shows significant upregulation of HER4 mRNA levels in treatment resistant tumours (left). This difference is even bigger within the Pseudo HER2 patient population (right). L) Boxplot of HER4 RRPA data shows no difference in protein abundance between treatment outcome groups. M) Boxplot of HER3 RPPA data (left) and HER3 mRNA data shows no difference between treatment outcome groups. * $p\text{-value} < 0.05$ ** $p\text{-value} < 0.01$

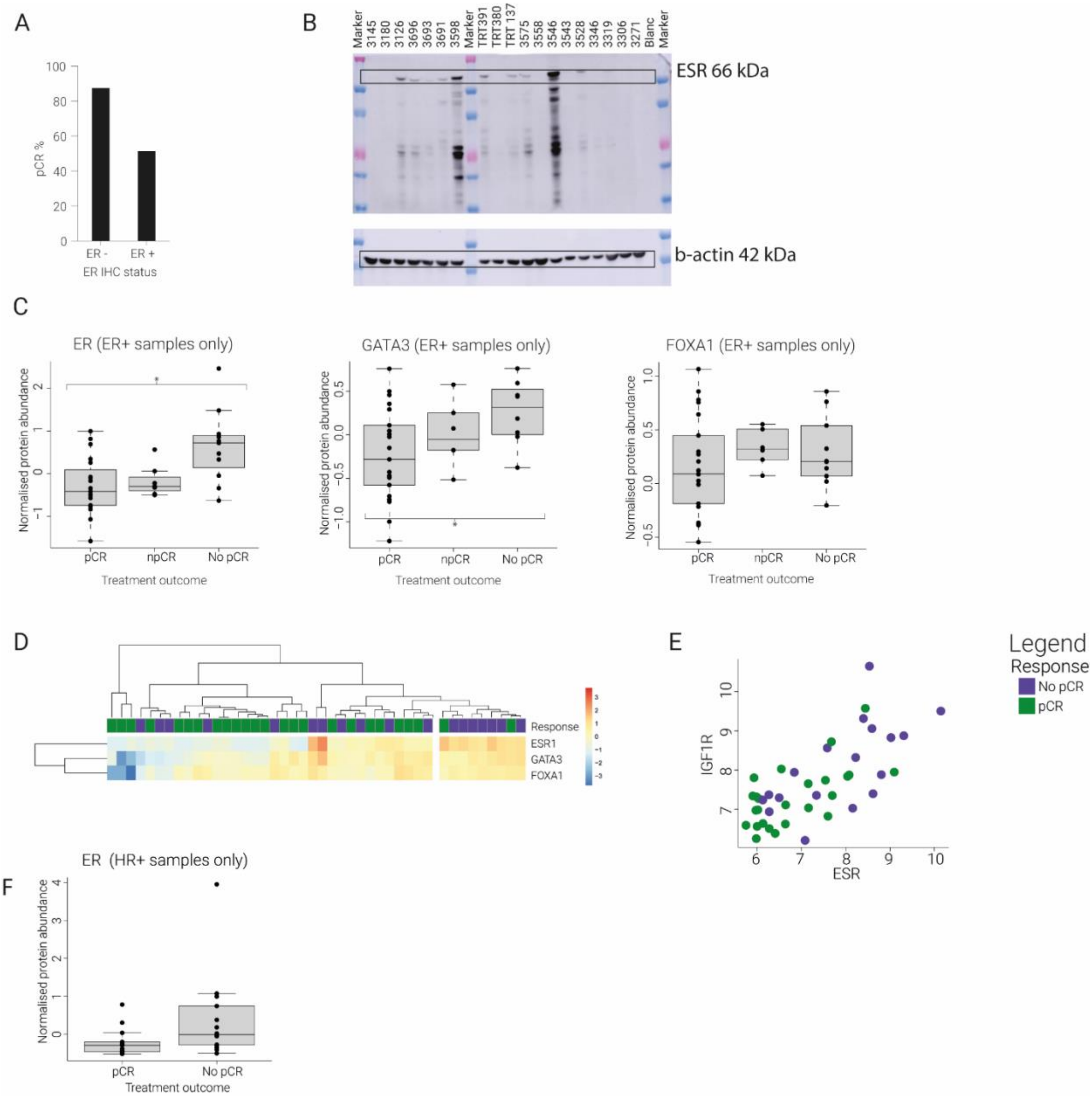

Supplementary Figure 3. ER signalling is associated with poor treatment response. Related to Figure 2. A) pCR rate (%) compared between the tumours with ER- status vs. ER+ as determined by IHC. B) WB data of ESR. Quantified bands indicated by box. ESR abundant was normalized to actin loading control. C) Boxplots of ER, GATA3 and FOXA1 expression amongst the three outcome groups for ER+ tumours only. D) Unsupervised clustering of mRNA expression of ESR, GATA3 and FOXA1 show clustering of treatment resistant patients with the highest ESR, GATA3 and FOXA1 mRNA levels. E) Correlation plot of ESR vs. IGF1R mRNA expression levels, * p-value < 0.05, ** p-value < 0.01. F) Boxplots of RPPA ER expression amongst the two outcome groups for hormone(HR)+ tumours only

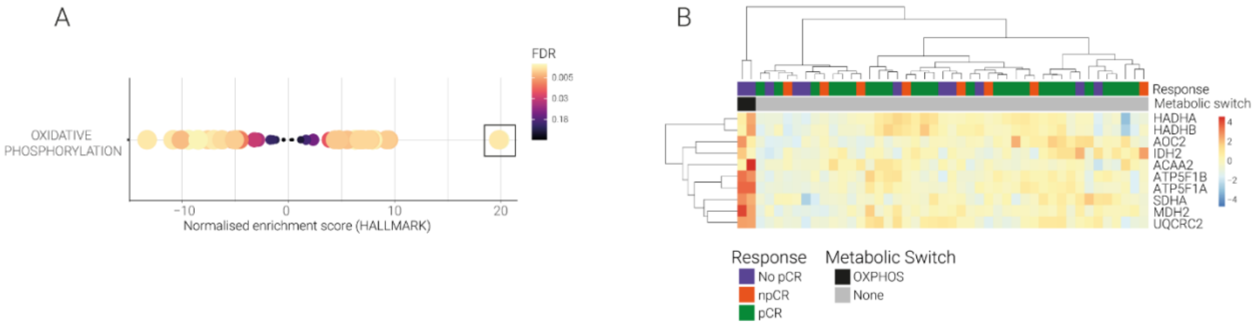

Supplementary Figure 4. Metabolic Switch preconditions tumours for therapy resistance. Related to Figure 3. A) GSEA analysis of oxidative phosphorylation Hallmark signature. Normalised enrichment score (NES) is plotted on the x-axis, dot size and colour represent the FDR. Tumour samples enclosed by black box are samples that have undergone a metabolic switch. B) Heatmap of unsupervised clustering of the top 10 most abundant mitochondrial proteins. Tumour samples that have undergone a metabolic switch are indicated in black. These grouped together and exhibited the highest abundance of mitochondrial proteins.

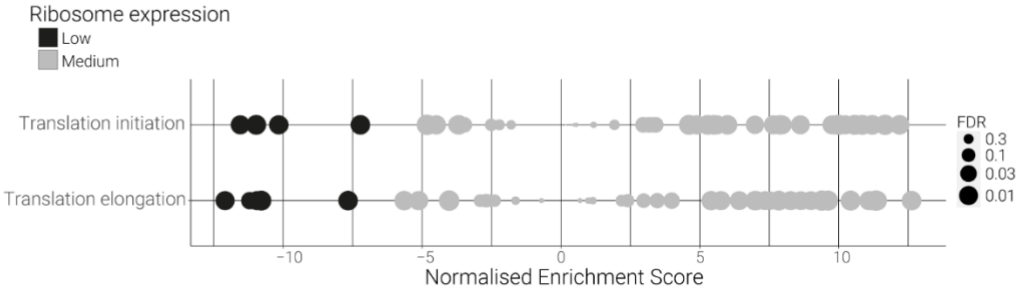

Supplementary Figure 5. UPR-induced cellular dormancy preconditions tumour to resist therapy. Related to Figure 4. Normalised enrichment scores of GSEA analysis of Reactome pathways showed reduced translation initiation and elongation amongst tumours with reduced ribosomal protein levels (shown in black). Dot size represents the GSEA FDR.

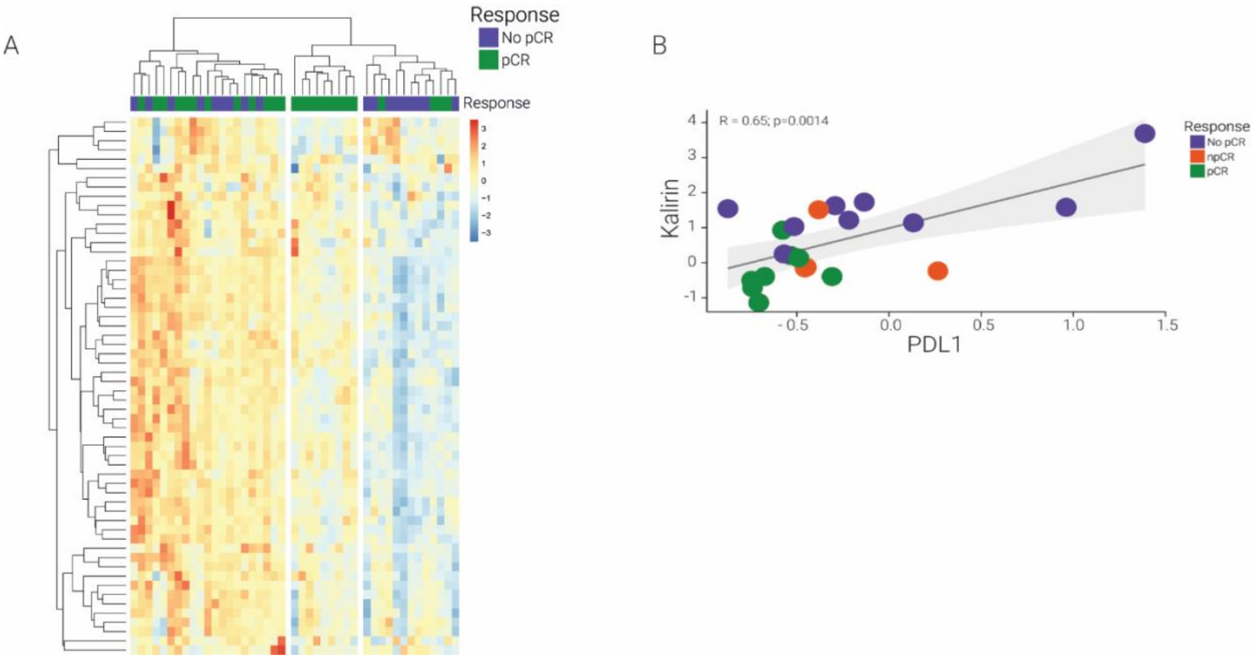

Supplementary Figure 6. Low immune cell infiltration levels correlate with poor treatment response. Related to Figure 5. A) Unsupervised clustering of immune cell marker expression shows clustering of treatment resistant patients with low immune cell marker mRNA levels. B) Kalirin expression correlates with PDL1 levels. Correlation plot comparing protein expression levels, normalized to internal pooled reference and log2 transformed, for all patients in the low-immune subset. Colour code represent patient pCR response. R = person correlation. Grey region represents the 95% confidence interval.
